# The effects of single and a combination of determinants of anaemia in the very old: results from the *TULIPS* consortium

**DOI:** 10.1186/s12877-021-02389-2

**Published:** 2021-08-09

**Authors:** Pin-Chun Wang, Jacobijn Gussekloo, Yasumichi Arai, Yukiko Abe, Jeanet W. Blom, Rachel Duncan, Carol Jagger, Ngaire Kerse, Carmen Martin-Ruiz, Leah Palapar, Wendy P. J. den Elzen

**Affiliations:** 1 Leiden University Medical Center, Master’s Vitality and Ageing, PO Box 9600, 2300 RC Leiden, the Netherlands; 2 Department of Clinical Chemistry and Laboratory Medicine, Leiden University Medical Center, PO Box 9600, 2300 RC Leiden, the Netherlands; 3 Department of Public Health and Primary Care, Leiden University Medical Center, PO Box 9600, 2300 RC Leiden, the Netherlands; 4 Department of Internal Medicine, Gerontology and Geriatrics Section, Leiden University Medical Center, PO Box 9600, 2300 RC Leiden, the Netherlands; 5 Center for Supercentenarian Medical Research, Keio University School of Medicine, Tokyo, Japan; 6 The Newcastle upon Tyne Hospitals NHS Foundation Trust, Freeman Hospital, Freeman Road, High Heaton, Newcastle upon Tyne, NE7 7DN UK; 7 Population Health Sciences Institute, Newcastle University, Newcastle upon Tyne, NE4 5PL UK; 8 Department of General Practice and Primary Health Care, University of Auckland, Auckland, 1072 New Zealand; 9 Biosciences Institute, Newcastle University, Newcastle upon Tyne, NE4 5PL UK; 10 Atalmedial Diagnostics Centre, Amsterdam, The Netherlands; 11 Department of Clinical Chemistry, Amsterdam UMC, Amsterdam, The Netherlands

**Keywords:** Vitamin B12/folate deficiency, Iron deficiency, Renal impairment, Inflammation, Low haemoglobin levels

## Abstract

**Background and objectives:**

Nutritional deficiencies, renal impairment and chronic inflammation are commonly mentioned determinants of anaemia. The aim of this study was to investigate the effects of these determinants, singly and in combination, on anaemia in the very old.

**Method:**

The TULIPS Consortium consists of four population-based studies in oldest-old individuals: Leiden 85-plus Study, LiLACS NZ, Newcastle 85+ study, and TOOTH. Five selected determinants (iron, vitamin B12, and folate deficiency; low estimated glomerular filtration rate (eGFR); and high C-reactive protein (CRP)) were summed. This sum score was used to investigate the association with the presence and onset of anaemia (WHO definition). The individual study results were pooled using random-effects models.

**Results:**

In the 2216 participants (59% female, 30% anaemia) at baseline, iron deficiency, low eGFR and high CRP were individually associated with the presence of anaemia. Low eGFR and high CRP were individually associated with the onset of anaemia.

In the cross-sectional analyses, an increase per additional determinant (adjusted OR 2.10 (95% CI 1.85–2.38)) and a combination of ≥2 determinants (OR 3.44 (95% CI 2.70–4.38)) were associated with the presence of anaemia. In the prospective analyses, an increase per additional determinant (adjusted HR 1.46 (95% CI 1.24–1.71)) and the presence of ≥2 determinants (HR 1.95 (95% CI 1.40–2.71)) were associated with the onset of anaemia.

**Conclusion:**

Very old adults with a combination of determinants of anaemia have a higher risk of having, and of developing, anaemia. Further research is recommended to explore causality and clinical relevance.

**Supplementary Information:**

The online version contains supplementary material available at 10.1186/s12877-021-02389-2.

## Background

The prevalence of anaemia, a reduction in the number of red blood cells, in older individuals is around 17% and increases with age to above 20% in those aged 85+ years [1]. Older persons with anaemia often report poor quality of life and complaints of fatigue and weakness. Anaemia in old age has been associated with a higher risk of falls, frailty, disability, depression, longer postoperative recovery, as well as lower physical and cognitive capacity, a higher rate of comorbidity, and an increased mortality risk [2–8].

Since anaemia is a highly prevalent and incident disorder in older persons with relevant consequences on functioning and survival, previous research has focussed on single causes of anaemia in old age, such as nutritional deficiencies (iron, vitamin B12, and folate deficiency) [9, 10], renal impairment and chronic inflammation [11–13].

Previous findings have shown that the combinations of determinants are associated with poor outcomes, such as multimorbidity and in-hospital mortality [14], and polypharmacy and drug-disease interactions [15]. In addition, laboratory profiles have shown an association with all-cause mortality risk in old age with increasing risk per laboratory abnormality [16]. We therefore hypothesised that an increment of determinants of anaemia increases the possibility (risk) of anaemia in old age. We investigated the effects of single, and a combination of determinants on anaemia among the very old cross-sectionally and prospectively in the *TULIPS* Consortium, a collaboration of five international cohorts from four studies in very old individuals.

## Methods

### Study population and procedures

The Towards Understanding Longitudinal International Older People Studies (*TULIPS*) Consortium was established in 2014 and is a collaboration of five cohorts from four studies in four countries: Leiden 85-plus Study in the Netherlands, LiLACS NZ in New Zealand, Newcastle 85+ study in the United Kingdom, and TOOTH in Japan. All are observational, prospective and population-based studies that aim to investigate consequences of health and disease determinants in very old individuals.

The Leiden 85-plus Study recruited 85-year-old inhabitants in Leiden, the Netherlands. From September 1997 through September 1999, 705 inhabitants reached the age of 85 years and were eligible for participation in the study. Subjects were visited annually (at ages 85–90 years for 5 years) at their place of residence for face-to-face interviews and cognitive tests. Blood samples were taken according to predefined protocols under non-fasting conditions. The medical history was obtained from the participants, or partners and caregivers in memory-impaired subjects. Pharmacy records were studied annually, and participants were also interviewed to evaluate the use of (over-the-counter) supplements. The Medical Ethical Committee of the Leiden University Medical Center approved the study, and informed consent was obtained from all subjects or their guardian for cognitively impaired subjects [17].

The Life and Living in Advanced Age (LiLACS NZ) is a population-based study of two cohorts: Māori and non-Māori in advanced age in New Zealand. Participants were living in the Bay of Plenty and were born between 1 January, 1925 and 31 December, 1925 for non-Māori (85 years) and between 1920 and 1930 for Māori (80–90 years old) [18, 19]. At baseline, participants were interviewed using a comprehensive questionnaire, conducted by trained interviewers, and had a health assessment conducted by a trained nurse. Blood samples were collected at baseline after an overnight fasting. Information about medication and supplement intake was collected. A simplified version of the questionnaire was made available for those unable to do the comprehensive one [18]. Ethical approval for the LiLACS NZ was granted by the Northern X Regional Ethics Committee (NTX 09/09/088) in December 2009 and written informed consent was obtained from all participants for each stage of the study [19].

The Newcastle 85+ study recruited individuals turning 85 years old in 2006 (born in 1921) and who were registered with a participating National Health Service (NHS) general practice in the areas of Newcastle upon Tyne or North Tyneside in the north-east of England. Older persons living in care homes and with disabilities and/or cognitive impairment were included. At baseline (2006/2007), participants underwent a detailed multidimensional health assessment by trained research nurses in their residence (own home or institution), containing questionnaires, measurements, function tests, a fasting blood sample, and a review of medical records held by the general practice [20, 21]. Participants were followed up for an assessment of health and functioning (including self-reported physical activity) at 18 months (1.5 years; wave 2), 36 (3 years; wave 3), and 60 months (5 years; wave 4) by research nurses at their usual place of residence [22, 23]. For the present analysis, data were available for wave 2 and wave 3. The study was approved by the Newcastle and North Tyneside local research ethics committee (06/Q0905/2). Written informed consent was obtained from all participants, otherwise from a caregiver or a relative for those with cognitive impairments [20].

The Tokyo Oldest Old Survey on Total Health (TOOTH) recruited a random sample of community-dwelling seniors aged 85 years or older in the city of Tokyo, Japan. The baseline comprehensive assessment consisted of an in-home interview, a self-administered questionnaire, and a medical/dental examination. Participants were invited to Keio University Hospital for biomedical assessments. For those who were unable to visit the study centre, home-based examinations were done through visits by a geriatrician and a dentist. Socio-demographic characteristics and diseases at baseline were collected. However, since not all determinants were collected in the first year in TOOTH, we defined 3-year follow-up as baseline data, and 6-year follow-up as follow-up variable. The study was approved by the ethical committee of the Keio University School of Medicine (N0. 20,070,047, Dec2007) [24]. Written informed consent was obtained either from the participants or by their proxy (normally a family member or caregiver) if necessary.

A flow chart of recruitment and schematic representation of data samples in the five cohorts is shown in Supplementary Figure 1.

### Study parameters/measures

#### Outcome

In all studies, haemoglobin was measured in anti-coagulated whole blood samples (ethylenediaminetetraacetic acid (EDTA)-coated tubes) using standard automated analysis systems. The methods and analysers are listed in Supplementary Table 2. Anaemia was defined by the World Health Organization (WHO) as haemoglobin < 13 g/dL in men and < 12 g/dL in women [25]. In the cross-sectional analyses, the presence of anaemia at baseline served as the outcome variable; in the prospective analyses, the onset of anaemia during follow-up was the outcome variable. When a new case of anaemia was identified, we assumed that the anaemia had developed halfway the period between two follow-up blood measurements.

#### Determinants of anaemia

Iron deficiency, vitamin B12 deficiency, folate deficiency, low estimated glomerular filtration rate (eGFR), and high C-reactive protein (CRP) were selected as single determinants of anaemia and were analysed to investigate their association with the presence and onset of anaemia.

Ferritin level was considered an indicator of iron status [26], with the cut-off of < 15 μg/L for women and < 20 μg/L for men as deficiency. Vitamin B12 deficiency was defined as < 150 pmol/L. [27, 28] Folate deficiency was defined using specific cut-offs in the five cohorts, which were serum folate level < 7 nmol/L for the Leiden 85-plus Study and TOOTH [28], red blood cell folate < 317 nmol/L for LiLACS NZ [19] and red blood cell folate < 340 nmol/L for the Newcastle 85+ study [21, 29]. The MDRD (Modification of Diet in Renal Disease) Study equation was adopted to calculate eGFR [30–32]. Low eGFR was defined as eGFR below 45 ml/min/1.73m^2^ [12]. C-reactive protein levels higher than 5 mg/L were defined as high CRP [33].

Determinants with abnormal values were defined as 1 and those within normal range as 0. The combination of determinants was calculated as the sum of abnormal determinants at baseline ranging 0–5. Participants with missing data on single determinants were considered as within the reference intervals (In total, 104 (4.7%) for ferritin; 161 (7.3%) for vitamin B12; 103 (4.6%) for folate; 4 (0.2%) for eGFR; 6 (0.3%) for CRP). Those with missing data of all determinants were excluded from the analyses (*n* = 4). Then, participants were divided into two groups based on the sum of the determinants: 0 to 1 vs. 2 and above determinants.

#### Other variables

As socio-demographic characteristics, we selected age, sex and institutionalisation, which were available in the five cohorts. Institutionalisation was regarded as living in a nursing home. Since only community-dwelling older adults were recruited in TOOTH, this variable was not applicable. Smoking was classified as non-smokers and past or current smokers. Multi-morbidity was composed of the presence or history of cerebrovascular accident (stroke), coronary heart disease (CHD), cancer and diabetes. It was stratified into 0 to 1 versus ≥2 as a binary variable in the analyses.

### Statistical analyses

First, each cohort was analysed cross-sectionally to investigate the association of single and a combination of determinants with the presence of anaemia. Then, the studies were analysed prospectively to investigate the effects of the single and the combination of the determinants on the development of incident anaemia. After analysing the studies separately, the data were pooled in a meta-analysis. We further explored the effects of using a cut-off of ferritin of 50 μg/L for iron deficiency, and a combination of significant single determinants as a sensitivity analysis.

Multivariate logistic regression analyses were used to calculate odds ratios (OR) and their 95% confidence intervals. We applied three models: model 1) unadjusted model; model 2) adjusted for socio-demographic characteristics (age, sex, institutionalisation) and smoking; and model 3) a fully adjusted model which included socio-demographic characteristics, smoking and multi-morbidity.

Prospective analyses were done in three studies for which follow-up data were available: the Leiden 85-plus Study, the Newcastle 85+ study and TOOTH. Participants with anaemia at baseline were excluded for the prospective analyses. Multivariate Cox Proportional-Hazards Models were performed, with adjustments for the aforementioned confounding variables.

After acquiring the individual cross-sectional results of the five cohorts and prospective results of the three studies, they were pooled using a two-stage individual participant data (IPD) meta-analysis [34]. Because of the clinical heterogeneity between the studies, random-effects models were applied.

The statistical analyses were performed with SPSS software (version 25) and Review Manager (RevMan). Version 5.3. Copenhagen: The Nordic Cochrane Centre, The Cochrane Collaboration, 2014. for meta-analyses.

## Results

Table 1 shows the baseline characteristics of the five cohorts. All participants in the Leiden 85-plus Study and the Newcastle 85+ study were 85 years old. The median age of the participants in the LiLACS NZ was 82 years for Māori and 85 years for non-Māori; and 90 years for TOOTH. Overall, among a total of 2216 participants, 59% were female and 30% had anaemia at baseline (21.0% for non-Māori, 21.7% for Māori in the LiLACS NZ, 28.5% in the Leiden 85-plus Study, 29.8% in the Newcastle 85+ study and 48.4% in TOOTH). Furthermore, subjects with a combination of ≥2 determinants accounted for 4.6% in TOOTH, 21.8% in Leiden 85-plus Study, 21.8% in the Newcastle 85+ study and 24.1% for non-Māori and 24.6% for Māori in the LiLACS NZ.

**Table 1:** Baseline Characteristics of Participants in Four Population-Based Studies of the Very-old^ab^

	Leiden 85-plus (*N* = 555)	LiLACS NZ Māori (*N* = 207)	LiLACS NZ Non-Māori (*N* = 357)	Newcastle 85+ (*N* = 752)	TOOTH (*N* = 345)
Age, median (IQR)	85	82 (80 to 84)	85 (84 to 85)	85.5 (85.2 to 85.8)	90 (89 to 92)
Sex, women n (%)	368 (66.3)	116 (56.0)	181 (50.7)	456 (60.6)	192 (55.7)
Institutionalisation^c^	101 (18.2)	2 (1.0)	9 (2.5)	62 (8.3)	NA
Anaemia^d^	158 (28.5)	45 (21.7)	75 (21.0)	224 (29.8)	167 (48.4)
Smoking					
Never	278 (50.9)	91 (44.4)	188 (52.7)	264 (35.2)	199 (59.2)
Past/Present	268 (49.1)	114 (55.6)	169 (47.4)	485 (64.8)	133 (39.6)
Multi-morbidity (≥2 diseases)^e^	84 (15.1)	54 (26.1)	121 (33.9)	88 (11.7)	26 (7.5)
Single Determinants^f^					
Iron Deficiency	41 (7.4)	5 (3.0)	20 (6.7)	77 (10.3)	56 (16.3)
Vitamin B12 Deficiency	85 (15.4)	23 (13.3)	48 (16.8)	131 (17.4)	14 (4.8)
Folate Deficiency	42 (7.6)	86 (45.5)	131 (40.2)	26 (3.5)	3 (1.0)
Low eGFR	111 (20.0)	37 (18.1)	49 (13.8)	234 (31.1)	21 (6.1)
High CRP	191 (34.4)	51 (24.8)	86 (24.2)	220 (29.3)	40 (11.7)
Sum of Combination of Abnormal Determinants					
0	226 (40.7)	71 (34.3)	121 (33.9)	262 (34.8)	229 (66.4)
1	208 (37.5)	85 (41.1)	150 (42.0)	326 (43.4)	100 (29.0)
2	103 (18.6)	36 (17.4)	76 (21.3)	132 (17.6)	14 (4.1)
3	16 (2.9)	15 (7.2)	9 (2.5)	30 (4.0)	2 (0.6)
4	2 (0.4)	0	0	2 (0.3)	0
5	0	0	1 (0.3)	0	0
Combination of Determinants (≥2)^g^	121 (21.8)	51 (24.6)	86 (24.1)	164 (21.8)	16 (4.6)

*Abbreviations*: *IQR* Interquartile range (25th–75th percentiles), *eGFR* Estimated glomerular filtration rate, *CRP* C-reactive protein, *NA* data not applicable (exclusion criteria in study design)

^a^ Only age was presented as median (interquartile range), other variables were presented as number (percentage)

^b^ LiLACS NZ contained two cohorts: Māori and non-Māori population; TOOTH: since not all determinants were collected at baseline, 3-year follow-up was defined as baseline, and 6-year follow-up as follow-up data

^c^ Living in a nursing home

^d^ Anaemia was defined as hemoglobin < 12 g/dL for women, < 13 g/dL for men according to the WHO criteria

^e^ Multi-morbidity was composed of stroke, coronary heart disease (CHD), cancer and diabetes. It was stratified into 0 to 1 or 2 and above as a binary variable

^f^ Iron deficiency was defined as ferritin < 20 μg/L for men, < 15 μg/L for women; vitamin B12 deficiency was < 150 pmol/L; folate deficiency was serum folate level < 7 nmol/L (Leiden 85-plus Study and TOOTH) or red blood cell folate < 317 nmol/L (LiLACS NZ), < 340 nmol/L (Newcastle 85+ study); low eGFR was < 45 mL/min/1.73 m^2^, eGFR was calculated using MDRD (Modification of Diet in Renal Disease) Study equation from the National Kidney Foundation. High CRP was > 5 mg/L.

^g^ All four studies included five determinants: iron, vitamin B12, folate deficiency, low eGFR, and high CRP

### Cross-sectional results per single determinant

Supplementary Table 3 shows the prevalence of anaemia at baseline, depending on the presence of single and a combination of determinants.

**Fig. 1:**
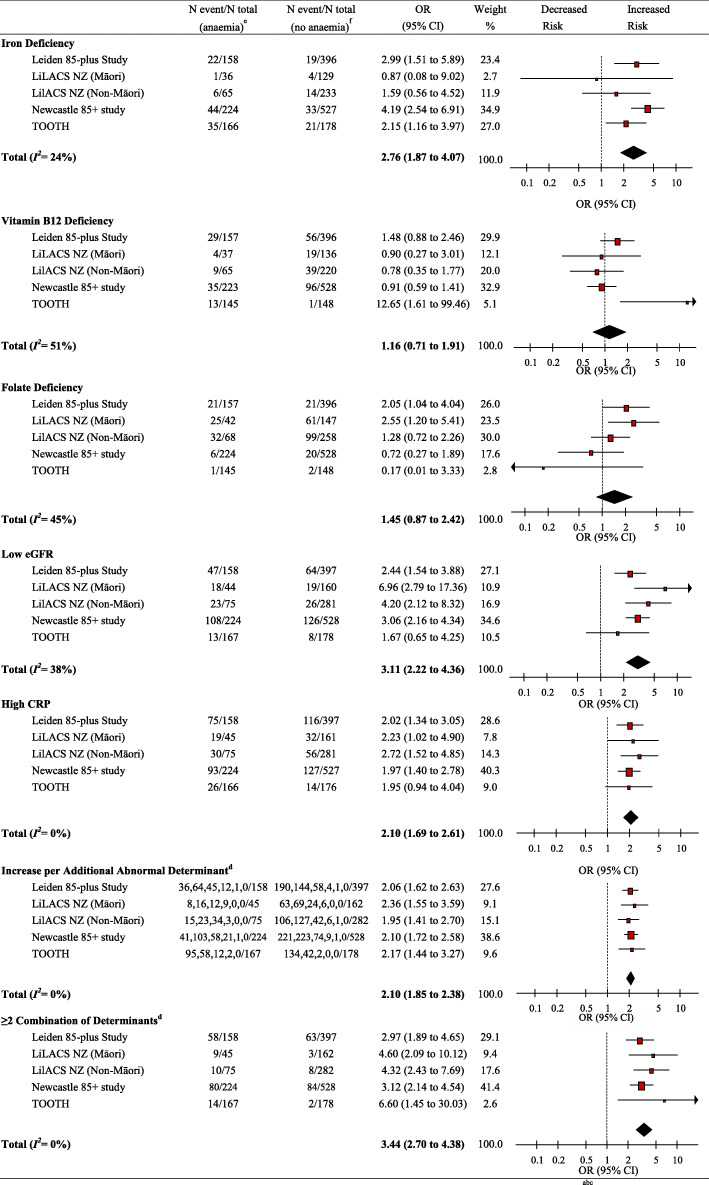
Cross-sectional Results: Meta-Analyses of Determinants of Anaemia at Baseline in Association with Presence of Anaemia ^abc.^ Abbreviations: OR, odds ratio; CI, confidence interval; eGFR, estimated glomerular filtration rate; CRP, C-reactive protein. ^a^ Iron deficiency was defined as ferritin < 20 μg/L for men, < 15 μg/L for women; vitamin B12 deficiency was < 150 pmol/L; folate deficiency was serum folate level < 7 nmol/L (Leiden 85-plus Study and TOOTH) or red blood cell folate < 317 nmol/L (LiLACS NZ) and < 340 nmol/L (Newcastle 85+ study); low eGFR was < 45 mL/min/1.73 m^2^, eGFR was calculated using MDRD (Modification of Diet in Renal Disease) Study equation from the National Kidney Foundation; high CRP was > 5 mg/L. ^b^ Results of fully adjusted model (model 3): adjusted for age, sex, institutionalisation, smoking and ≥ 2 multi-morbidity. Multi-morbidity was composed of stroke, coronary heart disease (CHD), cancer and diabetes. It was stratified into 0 to 1 or 2 and above as a binary variable. Leiden 85-plus Study: sex, institutionalisation, smoking and ≥ 2 multi-morbidity [stroke, coronary heart disease (CHD) excluding stroke, cancer, diabetes]; LiLACS NZ: age, sex, institutionalisation, smoking and ≥ 2 multi-morbidity [stroke (cerebrovascular accident (CVA), cardiovascular disease (CVD) excluding stroke, cancer, diabetes]; Newcastle 85+ study: age, sex, institutionalisation, smoking, ≥2 multi-morbidity (CVA, combined cardiac disease excluding CVA, cancer, diabetes); TOOTH: age, sex, smoking, ≥2 multi-morbidity (stroke, coronary heart disease (CHD), cancer, diabetes). ^c^ LiLACS NZ contained two cohorts: Māori and non-Māori population; TOOTH: since not all determinants were collected at baseline, 3-year follow-up was defined as baseline, and 6-year follow-up as follow-up data. ^d^ All four studies included five determinants: iron, vitamin B12, folate deficiency, low eGFR, and high CRP. ^e^ Population with determinant within total anemic population. ^f^ Population with determinant within total non-anemic population

Detailed results of unadjusted models and the two models with adjustments for confounders are displayed in Fig. 1 and Supplementary Table 4. After pooling the fully adjusted results of the five cohorts, the pooled adjusted OR for the presence of anaemia for participants with iron deficiency compared to their counterparts without iron deficiency was 2.76 (95% CI, 1.87 to 4.07, *I*^*2*^ = 24%). There was no evidence of an association between either vitamin B12 deficiency or folate deficiency and the presence of anaemia. Low eGFR and high CRP were associated with the presence of anaemia.

### Cross-sectional results for the combination of determinants

**Fig. 2:**
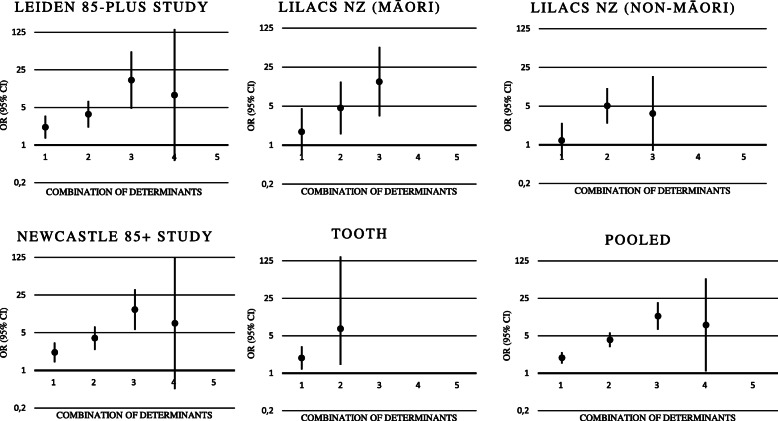
Cross-sectional Results: Trends of Odds Ratios of Combination of Determinants on the Presence of Anaemia ^ab^. Abbreviations: OR, odds ratio; CI, confidence interval. Participants with 0 determinant were the reference group. ^a^ Very low number of population size in some subgroups led to an inestimable odds ratio. ^b^ Results of fully adjusted model (model 3): adjusted for age, sex, institutionalisation, smoking and ≥ 2 multi-morbidity

The prevalence of 0, 1, 2, 3, 4 and 5 determinants for participants with and without anaemia are shown in Supplementary Table 5. Overall, between 17.8 and 56.9% of the participants with anaemia had a sumscore of 0. Per additional determinant the risk of the presence of anaemia was 2.10 (95% CI, 1.85 to 2.38, *I*^*2*^ = 0%, P trend< 0.001) (Figs. 1, 2). In addition, a combination of two or more abnormal determinants was significantly associated with the presence of anaemia in all the five cohorts, with a pooled adjusted OR of 3.44 (95% CI, 2.70 to 4.38, *I*^*2*^ = 0%; Fig. 1).

### Prospective results per single determinant

Supplementary Table 6 shows the incidence of anaemia at baseline, depending on the presence of single and a combination of determinants.

In the pooled analyses, participants with iron deficiency (Table 2), vitamin B12 deficiency, or folate deficiency (Table 2) did not have a higher risk of developing anaemia during follow-up. Low eGFR was associated with the onset of anaemia (pooled adjusted HR 1.62 95% CI, 1.04 to 2.54, *I*^*2*^ = 35%; Table 2). In addition, the pooled estimate of high CRP on the onset of anaemia was 1.40 (95% CI, 1.04 to 1.89, *I*^*2*^ = 0%; Table 2).

**Table 2:** Meta-Analyses of Single and Combination of Determinants of Anaemia in Association with Onset of Anaemia ^abc^

	N event/N total	N event/N total	HR	Weight
	(anaemia)^d^	(no anaemia)^e^	(95% CI)	%
**Iron Deficiency**				
Leiden 85-plus Study	6/98	11/262	1.48 (0.64 to 3.40)	30.8
Newcastle 85+ study	13/109	12/309	2.41 (1.35 to 4.32)	47.6
TOOTH	4/33	9/64	0.88 (0.30 to 2.53)	21.6
**Total (*I***^***2***^ **= 32%)**			**1.67 (0.96 to 2.90)**	100.0
**Vitamin B12 Deficiency**				
Leiden 85-plus Study	16/98	37/262	1.03 (0.59 to 1.79)	40.6
Newcastle 85+ study	25/109	49/309	1.35 (0.85 to 2.13)	59.4
TOOTH	0/28	1/54	f	0
**Total (*I***^***2***^ **= 0%)**			**1.21 (0.85 to 1.72)**	100.0
**Folate Deficiency**				
Leiden 85-plus Study	10/98	9/262	2.84 (1.45 to 5.54)	51.2
Newcastle 85+ study	2/109	12/309	0.56 (0.14 to 2.30)	31.9
TOOTH	1/28	1/54	1.20 (0.11 to 12.91)	17.0
**Total (*I***^***2***^ **= 54%)**			**1.46 (0.46 to 4.60)**	100.0
**Low eGFR**				
Leiden 85-plus Study	14/98	40/263	1.09 (0.61 to 1.94)	36.3
Newcastle 85+ study	41/109	64/309	1.97 (1.32 to 2.94)	52.7
TOOTH	3/33	0/64	2.41 (0.68 to 8.52)	11.0
**Total (*I***^***2***^ **= 35%)**			**1.62 (1.04 to 2.54)**	100.0
**High CRP**				
Leiden 85-plus Study	33/98	64/263	1.55 (1.00 to 2.42)	46.0
Newcastle 85+ study	29/109	63/308	1.27 (0.83 to 1.95)	48.1
TOOTH	3/33	3/63	1.45 (0.42 to 5.00)	5.9
**Total (*I***^***2***^ **= 0%)**			**1.40 (1.04 to 1.89)**	100.0
**Increase per Additional Abnormal Determinant**^**c**^				
Leiden 85-plus Study	41,37,18,2,0,0/98	139,91,30,2,0,0/263	1.35 (1.06 to 1.73)	44.0
Newcastle 85+ study	30,52,23,4,0,0/109	149,124,32,4, 0,0/309	1.58 (1.25 to 1.98)	50.3
TOOTH	23,9,1,0,0,0/33	50,14,0,0,0,0/64	1.28 (0.65 to 2.53)	5.7
**Total (*I***^***2***^ **= 0%)**			**1.46 (1.24 to 1.71)**	100.0
**≥2 Combination of Determinants**^**c**^				
Leiden 85-plus Study	20/98	33/263	1.87 (1.12 to 3.12)	41.1
Newcastle 85+ study	27/109	36/309	2.02 (1.30 to 3.13)	56.3
TOOTH	1/33	0/64	1.98 (0.25 to 15.53)	2.6
**Total (*I***^***2***^ **= 0%)**			**1.95 (1.40 to 2.71)**	100.0

*Abbreviations*: *HR* Hazard ratio, *CI* Confidence interval, *eGFR* Estimated glomerular filtration rate, *CRP* C-reactive protein

^a^ Iron deficiency was defined as ferritin < 20 μg/L for men, < 15 μg/L for women; vitamin B12 deficiency was < 150 pmol/L; folate deficiency was serum folate level < 7 nmol/L (Leiden 85-plus Study and TOOTH) or < 340 nmol/L (Newcastle 85+ Study); low eGFR was < 45 mL/min/1.73 m^2^, eGFR was calculated using MDRD (Modification of Diet in Renal Disease) Study equation from the National Kidney Foundation; high CRP was > 5 mg/L

^b^ Results of fully adjusted model (model 3): adjusted for age, sex, institutionalisation, smoking and ≥ 2 multi-morbidity. Multi-morbidity was composed of stroke, coronary heart disease (CHD), cancer and diabetes. It was stratified into 0 to 1 or 2 and above as a binary variable. Leiden 85-plus Study: sex, institutionalisation, smoking and ≥ 2 multi-morbidity [stroke, coronary heart disease (CHD) excluding stroke, cancer, diabetes]; Newcastle 85+ study: age, sex, institutionalisation, smoking, ≥2 multi-morbidity (CVA, combined cardiac disease excluding CVA, cancer, diabetes); TOOTH: age, sex, smoking, ≥2 multi-morbidity (stroke, coronary heart disease (CHD), cancer, diabetes)

^c^ LiLACS NZ did not have follow-up data for hemoglobin; TOOTH: since not all determinants were collected at baseline, 3-year follow-up was defined as baseline, and 6-year follow-up as follow-up data. All three studies included five determinants: iron, vitamin B12, folate deficiency, low eGFR, and high CRP

^d^ Population with determinant within total anemic population during follow-up

^e^ Population with determinant within total non-anemic population during follow-up

^f^ A population size of zero led to an inestimable hazard ratio

### Prospective results for the combination of determinants

**Fig. 3:**
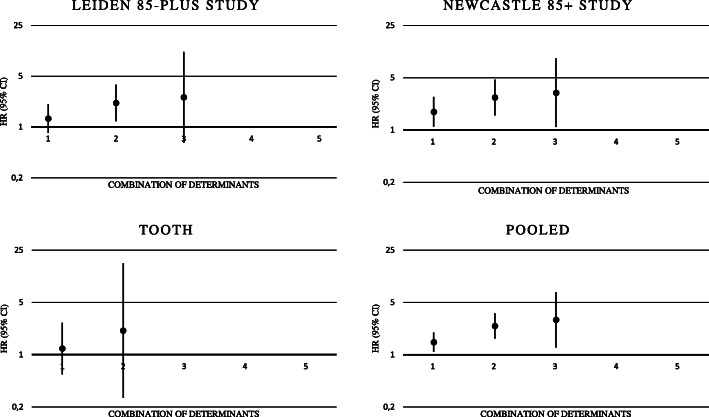
Prospective Results: Trends of Hazard Ratios of Combination of Determinants on the Onset of Anaemia ^ab^. Abbreviations: HR, hazard ratio; CI, confidence interval. Participants with 0 determinants were the reference group. ^a^ Very low number of population size in some subgroups led to an inestimable odds ratio. ^b^ Results of fully adjusted model (model 3): adjusted for age, sex, institutionalisation, smoking and ≥ 2 multi-morbidity

Per additional determinant the risk of developing anaemia was statistically significant in the Leiden 85-plus Study (HR 1.35 (95% CI, 1.06 to 1.73) and the Newcastle 85+ study (HR 1.58 (95% CI, 1.25 to 1.98) (Table 2), but not for TOOTH. The pooled estimate of the effect on the development of anaemia was 1.46 (95% CI, 1.24 to 1.71, *I*^*2*^ = 0%, P trend< 0.001) per additional determinant (Table 2, Fig. 3).

Participants with a combination of ≥2 determinants had a statistically significant 1.87-fold higher risk of developing anaemia compared to those with 0 or 1 determinant (95% CI, 1.12 to 3.12) in the Leiden 85-plus Study and Newcastle 85+ study (HR (95% CI), 2.02 (1.30 to 3.13)), but not in TOOTH. The pooled effect estimate for the onset of anaemia was 1.95 (95% CI, 1.40 to 2.7165, *I*^*2*^ = 0%; Table 2, Supplementary Table 7).

### Sensitivity analyses

Similar results were obtained when a cut-off of 50 μg/L was used to define iron deficiency (Supplementary Tables 8, 9, 10, 11 and 12).

We further explored the effects of a combination of single determinants (iron deficiency, low eGFR and high CRP) that were individually associated with the presence or onset of anaemia, as a sensitivity analysis.

In the cross-sectional analyses, the pooled estimate for the OR on the presence of anaemia was 2.64 (95% CI, 2.22 to 3.14, *I*^*2*^ = 15%) per additional determinant. In addition, compared to participants with 0 or 1 determinant, the pooled adjusted OR for a combination of ≥2 of the three significant determinants (iron deficiency, low eGFR and high CRP) on the presence of anaemia was 4.53 (95% CI, 2.66 to 7.72, *I*^*2*^ = 50%).

In the prospective analyses, per additional determinant, the risk of development of anaemia increased 1.54 fold (pooled adjusted HR (95% CI), 1.54 (1.24 to 1.92), *I*^*2*^ = 11%). In addition, participants with a combination of ≥2 significant determinants had a 1.99-fold higher risk of developing anaemia compared to those with 0 or 1 determinant (pooled adjusted HR 1.99 95% CI, 1.32 to 3.02, *I*^*2*^ = 0%).

## Discussion

### Principal findings

In this study, we found that very old adults with iron deficiency, low eGFR and high CRP at baseline had a higher risk of having anaemia. A combination of two or more determinants at baseline was associated with the presence of anaemia. Moreover, low eGFR, high CRP and a combination of two and above determinants were associated with the development of anaemia during follow-up.

### Comparison with other studies

This study confirmed the well-known association of iron deficiency and the presence of anaemia [35]. Surprisingly, iron deficiency was not associated with anaemia prospectively [36, 37]. Comparable with previous studies in other settings, low eGFR and high CRP were also associated with both the presence and the development of anaemia in this study [38–40].

We found an increased risk of folate deficiency on anaemia in the Leiden 85-plus Study that corresponded to the previous conclusion in 2008 [27]. However, in the pooled analysis in the *TULIPS* Consortium, we found no evidence that vitamin B12 and folate deficiency were associated with the presence or onset of anaemia. This is in line with earlier systematic reviews and meta-analyses showing no effect of treatment with vitamin B12 or folic acid on haematological parameters in older individuals [28].

The prevalence of anaemia in TOOTH was almost double that of the other four cohorts but the percentages of single determinants were half. This is in accordance to previous research stating that the frequency of anaemia in Japan is particularly high compared to other countries; moreover, other causes of anaemia such as myelodysplastic syndromes in Japan may be an important determinant for future studies [41].

### Strengths

First, having access to original datasets enabled us to conduct an individual participant data (IPD) meta-analysis to standardize definitions and analyses. In addition, the two-stage method allowed us present the individual study data and to explicitly show the differences in cohorts, and apply well-described standard meta-analysis methods [34]. Second, in the *TULIPS* Consortium, five cohorts in the very old population were combined which resulted in a large number of very old participants from across the world. This means our results can be extrapolated to a large and diverse group of older individuals worldwide.

### Weaknesses & limitations

We recognize several limitations in our study. First, no full diagnostic work-up for anaemia was performed. Still, our primary analyses are based on measurements of iron status, vitamin B12, folate, CRP and creatinine, which are direct measures or surrogates for the most commonly mentioned causes of anaemia in older persons [1]. These measures are also used in clinical practice to further investigate the cause of anaemia. In addition, we incorporated major clinical conditions such as cardiovascular disease and cancer as important indicators of clinical status, which we accounted for in the analyses. Second, information on serum iron, iron saturation, MCV or other blood counts were not available in all studies. Third, we adjusted for a limited number of comorbidities (cardiovascular disease, cancer and diabetes), as information on other comorbidities was not present in all studies. Residual confounding by e.g. liver disease or gastro-intestinal bleeding may have led to an overestimation of the true associations. Fourth different methods and analysers were applied for the laboratory measurements in the five cohorts. However, the laboratory methods were consistent within each study and were analysed on a study level, which was likely not to have resulted in systematic error, making the effect of differences in laboratory measurements a minor concern. Fifth, small numbers in some subgroups led to inestimable results which could not be pooled in the meta-analyses.

In addition, participants with missing data on single determinants were assumed to be within normal range which could have also led to an underestimation of the current results [42]. Last, the determinants were measured once at baseline. Patients who had transient deficiency, who developed abnormality over time, or who reverted to normal levels probably due to supplement use during follow-up may have been misclassified.

## Conclusions

This study showed that a combination of determinants of anaemia is associated with the presence and development of anaemia in the very old. Since anaemia in the very old is common and associated with poor outcomes [43], health professionals need to be aware that individuals over 85 years of age with a combination of determinants of anaemia present with a high risk of prevalent and incident anaemia. However, further research is needed to explore the underlying pathophysiological mechanisms, either on the specific combinations of determinants, or on the effects within different ethnicities and in younger populations.

## Supplementary Information

**Additional file 1: Figure S1.** Recruitment Flowchart and Schematic Representation of Data Samples Used in the Four Studies. **Table S2.** Laboratory Methods and Analysers of the Four Studies. **Table S3.** Prevalence of Anaemia at Baseline, Depending on the Presence of Single and a Combination of Determinants. **Table S4.** Cross-sectional Results: Single and a Combination of Determinants of Anaemia in Association with the Presence of Anaemia at Baseline in the Four Studies (Crude and Two Adjusted Models). **Table S5.** The prevalence of 0, 1, 2, 3, 4 and 5 (out of 5) abnormal determinants for participants with and without anemia at baseline in four studies. **Table S6.** Incidence of Anaemia from Age 85 Years Onwards, depending on the presence of single and a combination of determinants. **Table S7.** Prospective Results: Single and a Combination of Determinants at Baseline in Association with the Onset of Anaemia during Follow-up in Three Studies (Crude and Two Adjusted Models). **Table S8.** The prevalence of iron deficiency and the combination of abnormal determinants at baseline. in the four studies using two different cut-offs for serum ferritin to define iron deficiency **Table S9.** Cross-sectional results: the association between iron deficiency, using two cut-offs for ferritin concentration, and the presence of anaemia at baseline in the four studies. **Table S10.** Meta-Analyses: Iron Deficiency (using two Cut-offs for ferritin concentration), Combination of Determinants of Anaemia at Baseline in Association with Presence of Anaemia. **Table S11.** Prospective results: the association between iron deficiency, using two cut-offs for ferritin concentration, and the onset of anaemia at baseline in three studies. **Table S12.** Meta-Analyses: Iron Deficiency (using two Cut-offs for ferritin concentration) and Combination of Determinants of Anaemia in Association with Onset of Anaemia in Three Studies.

## Data Availability

The datasets used and/or analysed during the current study are available from the corresponding author on reasonable request.

## Abbreviations

eGFR: Estimated glomerular filtration rate
CRP: C-reactive protein
WHO: World Health Organization
OR: Odds ratio
CI: Confidence interval
HR: Hazard ratio
*TULIPS*: Towards Understanding Longitudinal International Older People Studies
LiLACS NZ: Life and Living in Advanced Age
TOOTH: Tokyo Oldest Old Survey on Total Health
NHS: National Health Service
EDTA: Ethylenediaminetetraacetic acid
MDRD: Modification of Diet in Renal Disease
CHD: Coronary heart disease
IPD: Individual participant data
