# Supplementary material for: The effects of single and a combination of determinants of anaemia in the very old: results from the TULIPS consortium
Source: BMC Geriatr. 2021 Aug 9;21:457. doi: 10.1186/s12877-021-02389-2 (PMC8351428; doi:10.1186/s12877-021-02389-2)
Supplement: Supplementary file 1 — Additional file 1: Figure S1. Recruitment Flowchart and Schematic Representation of Data Samples Used in the Four Studies. Table S2. Laboratory Methods and Analysers of the Four Studies. Table S3. Prevalence of Anaemia at Baseline, Depending on the Presence of Single and a Combination of Determinants. Table S4. Cross-sectional Results: Single and a Combination of Determinants of Anaemia in Association with the Presence of Anaemia at Baseline in the Four Studies (Crude and Two Adjusted Models). Table S5. The prevalence of 0, 1, 2, 3, 4 and 5 (out of 5) abnormal determinants for participants with and without anemia at baseline in four studies. Table S6. Incidence of Anaemia from Age 85 Years Onwards, depending on the presence of single and a combination of determinants. Table S7. Prospective Results: Single and a Combination of Determinants at Baseline in Association with the Onset of Anaemia during Follow-up in Three Studies (Crude and Two Adjusted Models). Table S8. The prevalence of iron deficiency and the combination of abnormal determinants at baseline. in the four studies using two different cut-offs for serum ferritin to define iron deficiency Table S9. Cross-sectional results: the association between iron deficiency, using two cut-offs for ferritin concentration, and the presence of anaemia at baseline in the four studies. Table S10. Meta-Analyses: Iron Deficiency (using two Cut-offs for ferritin concentration), Combination of Determinants of Anaemia at Baseline in Association with Presence of Anaemia. Table S11. Prospective results: the association between iron deficiency, using two cut-offs for ferritin concentration, and the onset of anaemia at baseline in three studies. Table S12. Meta-Analyses: Iron Deficiency (using two Cut-offs for ferritin concentration) and Combination of Determinants of Anaemia in Association with Onset of Anaemia in Three Studies. [file 12877_2021_2389_MOESM1_ESM.docx]

Supplementary material

| **Leiden 85-plus Study** | **LiLACS NZ** | **Newcastle 85+ study** | **TOOTH** |
| --- | --- | --- | --- |
| 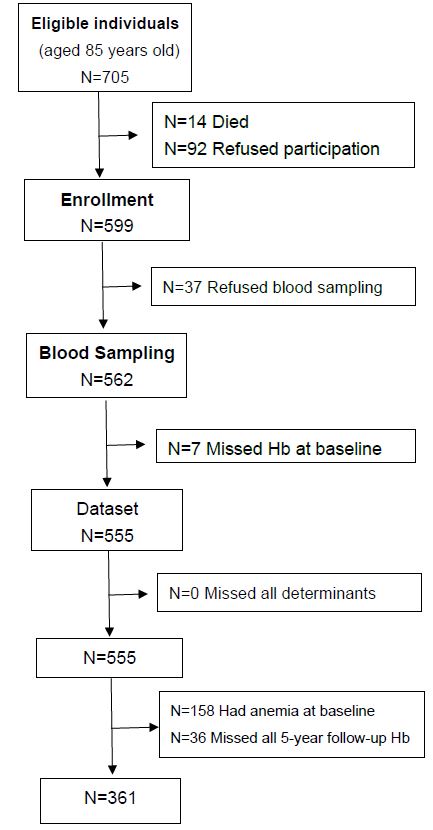 | 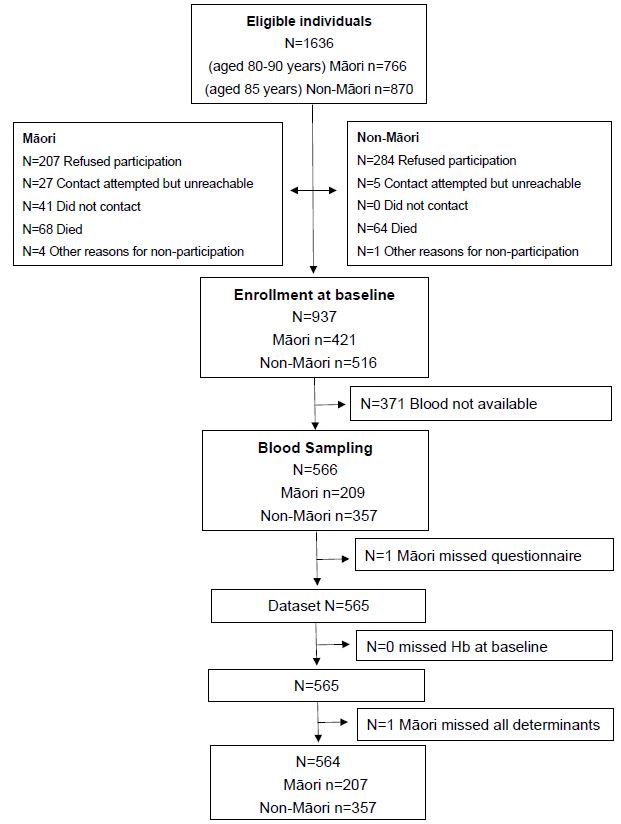 | 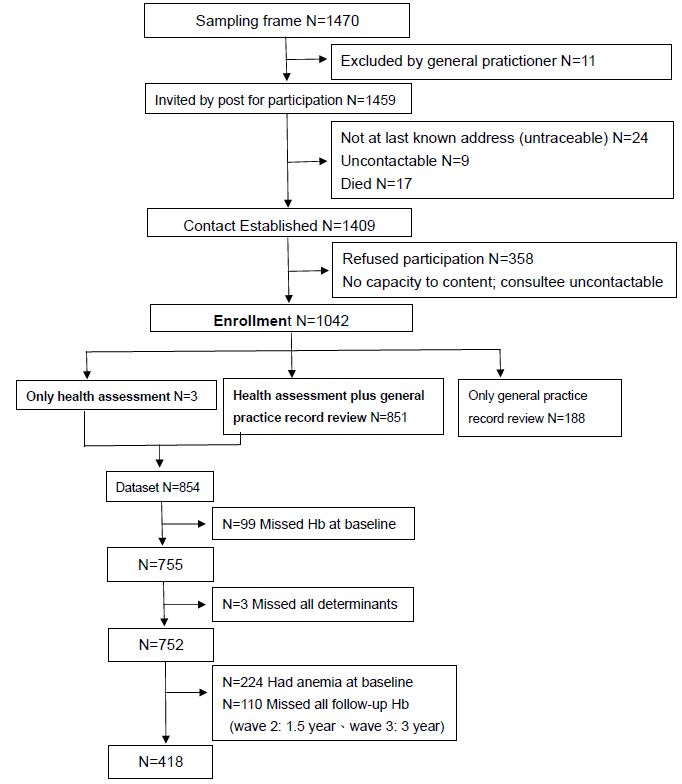  Cross-sectional Analyses | 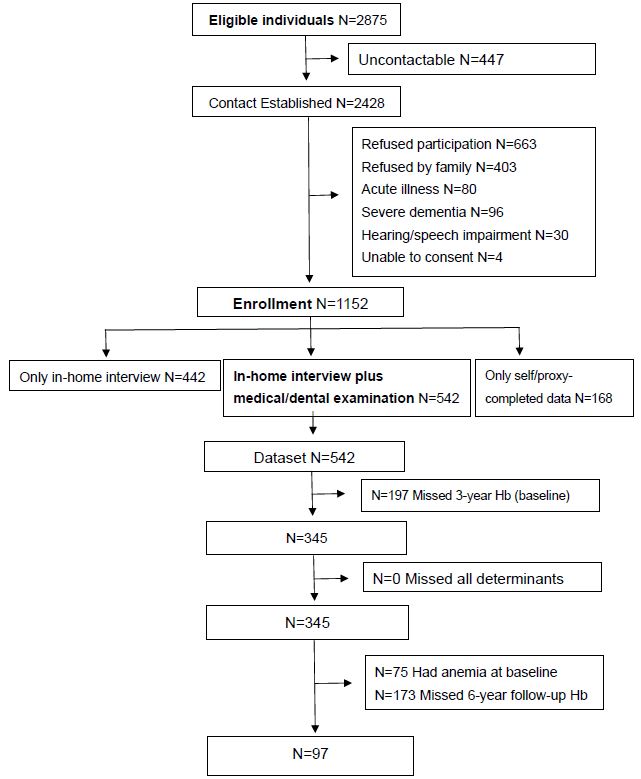  Prospective Analyses |
| **Supplementary Figure 1**. Recruitment Flowchart and Schematic Representation of Data Samples Used in the Four Studies | | | |

| **Supplementary Table 2.** Laboratory Methods and Analysers of the Four Studies | | | | |
| --- | --- | --- | --- | --- |
|  | **Leiden 85-plus Study** | **LiLACS NZ** | **Newcastle 85+ Study** | **TOOTH** |
| Hemoglobin | Coulter Counter, Coulter Electronics, Hialeah, USA | Photometric measurement  (unicel dxh 800 coulter cellular analysis system, beckman coulter, inc. Chaska, mn, usa) | Tosoh Eurogenetics automated HLC-723G7 HPLC analyser | Flow cytometry using a semiconductor laser, a sheath flow DC detection method, SLS-HGB, RBC pulse height detection method (XE-2100, SYSMEX CORPORATION, Japan) |
| Serum Ferritin | Immunologic assay (E170, Roche,  Almere, the Netherlands). | Chemiluminescence (Cobas 8000, module c602, Roche Diagnostics, Manheim, Germany) | Immunoradiometric assay (Ferritin Mab; ICN Pharmaceuticals | CLEIA  (LUMIPULSEL2400, BFUJIREBIO INC., Japan) |
| Serum Vitamin B12 | Dual count solid phase no boil assay (diagnostic products corporation, los angeles, california, usa). | Chemiluminescence (unicel dxi 800 Immunoassay System, Beckman Coulter, Inc., Chaska, MN, USA) | Chemiluminescence  (microparticle immunoassay on an abbott architect analyser) | CLEIA  (UniCel DxI 800, Beckman Coulter, Inc., United States of America, USA) |
| Serum Folate/ Red Blood Cell Folate | Dual count solid phase no boil assay (diagnostic products corporation, los angeles, california, usa) | [**Red Blood Cell folate**]  Chemiluminescence (unicel dxi 800 Immunoassay System, Beckman  Coulter, Inc., Chaska, MN, USA) | [**Red Blood Cell folate**] chemiluminescence (Microparticle Immunoassay on an Abbott ARCHITECT analyser) | CLEIA  (UniCel DxI 800, Beckman Coulter, Inc., United States of America, USA) |
| Serum Creatinine | Jaffe method (Hitachi 747, Tokyo, Japan) | Automated HPLC Abbott Architect assay | Tosoh Eurogenetics automated HLC-723G7 HPLC analyser | Enzymatic method  (Bio Majesty (JCA-BM6010), JEOL Ltd., Japan) |
| C-reactive protein (CRP) | Immunoturbidimetric assay, Hitachi 747 automated analyser  (Hitachi, Tokyo, Japan) | Immunoturbidimetric assay (Roche, Auckland, New Zealand) | High-sensitivity immunoassay (cardiophase, Dade Behring, Deerfield, IL) in Behring Nephelometer. | Latex turbidimetric immunoassay (JCA-BM8060, JEOL Ltd., Japan) |

| **Supplementary Table 3** Prevalence of Anaemia at Baseline, Depending on the Presence of Single and a Combination of Determinants | | | | | | | |
| --- | --- | --- | --- | --- | --- | --- | --- |
|  | | | Index group  N (%)^a^ | Prevalence of anaemia at baseline in index group, N (%)^ab^ | Prevalence of anaemia at baseline in reference group, N (%)^c^ | Crude OR (95% CI)^d^ | P value (chi squared test)^e^ |
| **Iron deficiency** ^f^ | | |  |  |  |  |  |
|  | Leiden 85-plus Study | | 41/554 (7.4) | 22/41 (53.7) | 136/513 (26.5) | 3.21 (1.69 to 6.11) | <0.001 |
|  | LiLACS NZ (Māori) | | 5/165 (3.0) | 1/5 (20.0) | 35/160 (21.9) | 0.89 (0.10 to 8.25) | 0.92 |
|  | LilACS NZ (Non-Māori) | | 20/298 (6.7) | 6/20 (30.0) | 59/278 (21.2) | 1.59 (0.59 to 4.32) | 0.36 |
|  | Newcastle 85+ study | | 77/751 (10.3) | 44/77 (57.1) | 180/674 (26.7) | 3.66 (2.26 to 5.93) | <0.001 |
|  | TOOTH | | 56/344 (16.3) | 35/56 (62.5) | 131/288 (45.5) | 2.00 (1.11 to 3.60) | 0.02 |
| **Vitamin B12 deficiency** | | |  |  |  |  |  |
|  | Leiden 85-plus Study | | 85/553 (15.4) | 29/85 (34.1) | 128/468 (27.4) | 1.38 (0.84 to 2.25) | 0.20 |
|  | LiLACS NZ (Māori) | | 23/173 (13.3) | 4/23 (17.4) | 33/150 (22.0) | 0.75 (0.24 to 2.35) | 0.62 |
| … | LilACS NZ (Non-Māori) | | 48/285 (16.8) | 9/48 (18.8) | 56/237 (23.6) | 0.75 (0.34 to 1.63) | 0.46 |
|  | Newcastle 85+ study | | 131/751 (17.4) | 35/131 (26.7) | 188/620 (30.3) | 0.84 (0.55 to 1.28) | 0.41 |
|  | TOOTH | | 14/293 (4.8) | 13/14 (92.9) | 132/279 (47.3) | 14.48 (1.87 to 112.2) | <0.001 |
| **Folate deficiency** | | |  |  |  |  |  |
|  | Leiden 85-plus Study | | 42/553 (7.6) | 21/42 (50.0) | 136/511 (26.6) | 2.76 (1.46 to 5.21) | 0.002 |
|  | LiLACS NZ (Māori) | | 86/189 (45.5) | 25/86 (29.1) | 17/103 (16.5) | 2.07 (1.03 to 4.17) | 0.04 |
|  | LilACS NZ (Non-Māori) | | 131/326 (40.2) | 32/131 (24.4) | 36/195 (18.5) | 1.43 (0.83 to 2.45) | 0.19 |
|  | Newcastle 85+ study | | 26/752 (3.5) | 6/26 (23.1) | 218/726 (30.0) | 0.70 (0.28 to 1.77) | 0.45 |
|  | TOOTH | | 3/293 (1.0) | 1/3 (33.3) | 144/290 (49.7) | 0.51 (0.05 to 5.65) | 0.57 |
| **Low eGFR** | | |  |  |  |  |  |
|  | Leiden 85-plus Study | | 111/555 (20.0) | 47/111 (42.3) | 111/444 (25.0) | 2.20 (1.43 to 3.40) | <0.001 |
|  | LiLACS NZ (Māori) | | 37/204 (18.1) | 18/37 (48.6) | 26/167 (15.6) | 5.14 (2.38 to 11.08) | <0.001 |
|  | LilACS NZ (Non-Māori) | | 49/356 (13.8) | 23/49 (46.9) | 52/307 (16.9) | 4.34 (2.30 to 8.19) | <0.001 |
|  | Newcastle 85+ study | | 234/752 (31.1) | 108/234 (46.2) | 116/518 (22.4) | 2.97 (2.14 to 4.13) | <0.001 |
|  | TOOTH | | 21/345 (6.1) | 13/21 (61.9) | 154/324 (47.5) | 1.79 (0.72 to 4.45) | 0.20 |
| **High CRP** | | |  |  |  |  |  |
|  | Leiden 85-plus Study | | 191/555 (34.4) | 75/191 (39.3) | 83/364 (22.8) | 2.19 (1.50 to 3.20) | <0.001 |
|  | LiLACS NZ (Māori) | | 51/206 (24.8) | 19/51 (37.3) | 26/155 (16.8) | 2.95 (1.45 to 5.97) | 0.002 |
|  | LilACS NZ (Non-Māori) | | 86/356 (24.2) | 30/86 (34.9) | 45/270 (16.7) | 2.68 (1.55 to 4.63) | <0.001 |
|  | Newcastle 85+ study | | 220/751 (29.3) | 93/220 (42.3) | 131/531 (24.7) | 2.24 (1.60 to 3.12) | <0.001 |
|  | TOOTH | | 40/342 (11.7) | 26/40 (65.0) | 140/302 (46.4) | 2.15 (1.08 to 4.28) | 0.03 |
| **Sum of Abnormal Determinants**^g^ | | | Index group  N (%) | Prevalence of anaemia at baseline in index group, N (%) | Prevalence of anaemia at baseline in reference group, N (%) | Crude odds ratio (95% CI) | P value (Logistic Regression)^e^ |
| Leiden 85-plus Study | | 0 | 226/555 (40.7) | 36/226 (15.9) | Reference | 1 |  |
|  | | 1 | 208/555 (37.5) | 64/208 (30.8) | 36/226 (15.9) | 2.35 (1.48 to 3.72) | <0.001 |
|  | | 2 | 103/555 (18.6) | 45/103 (43.7) |  | 4.10 (2.42 to 6.94) | <0.001 |
|  | | 3 | 16/555 (2.9) | 12/16 (75.0) |  | 15.83 (4.83 to 51.86) | <0.001 |
|  | | 4 | 2/555 (0.4) | 1/2 (50.0) |  | 5.28 (0.32 to 86.33) | 0.243 |
|  | | 5 | 0/555 (0) | 0/0 |  | - | - |
| LiLACS NZ (Māori) | | 0 | 71/207 (34.3) | 8/71 (11.3) | Reference | 1 |  |
|  | | 1 | 85/207 (41.1) | 16/85 (18.8) | 8/71 (11.3) | 1.83 (0.73 to 4.56) | 0.20 |
|  | | 2 | 36/207 (17.4) | 12/36 (33.3) |  | 3.94 (1.43 to 10.82) | 0.01 |
|  | | 3 | 15/207 (7.2) | 9/15 (60.0) |  | 11.81 (3.32 to 41. 99) | <0.001 |
|  | | 4 | 0/207 (0) | 0/0 |  | - | - |
|  | | 5 | 0/207 (0) | 0/0 |  | - | - |
| LilACS NZ (Non-Māori) | | 0 | 121/357 (33.9) | 15/121 (12.4) | Reference | 1 |  |
|  | | 1 | 150/357 (42.0) | 23/150 (15.3) | 15/121 (12.4) | 1.28 (0.64 to 2.58) | 0.49 |
|  | | 2 | 76/357 (21.3) | 34/76(44.7) |  | 5.72 (2.83 to 11.58) | <0.001 |
|  | | 3 | 9/357 (2.5) | 3/9 (33.3) |  | 3.53 (0.80 to 15.64) | 0.10 |
|  | | 4 | 0/357 (0) | 0/0 |  | - ^h^ | - |
|  | | 5 | 1/357 (0.3) | 0/1 (0) |  | - | - |
| Newcastle 85+ study | | 0 | 262/752 (34.8) | 41/262 (15.6) | Reference | 1 |  |
|  | | 1 | 326/752 (43.4) | 103/326 (31.6) | 41/262 (15.6) | 2.49 (1.66 to 3.74) | <0.001 |
|  | | 2 | 132/752 (17.6) | 58/132 (43.9) |  | 4.23 (2.62 to 6.82) | <0.001 |
|  | | 3 | 30/752 (4.0) | 21/30 (70.0) |  | 12.58 (5.38 to 29.40) | <0.001 |
|  | | 4 | 2/752 (0.3) | 1/2 (50.0) |  | 5.39 (0.33 to 87.91) | 0.24 |
|  | | 5 | 0/752 (0) | 0/0 |  | - | - |
| TOOTH | | 0 | 229/345 (66.4) | 95/229 (41.5) | Reference | 1 |  |
|  | | 1 | 100/345 (29.0) | 58/100 (58.0) | 95/229 (41.5) | 1.95 (1.21 to 3.14) | 0.006 |
|  | | 2 | 14/345 (4.1) | 12/14 (85.7) |  | 8.46 (1.85 to 38.69) | 0.006 |
|  | | 3 | 2/345 (0.6) | 2/2 (100.0) |  | - | - |
|  | | 4 | 0/345 (0) | 0/0 (0) |  | - | - |
|  | | 5 | 0/345 (0) | 0/0 (0) |  | - | - |
| **Combination of Determinants**^g^ | | | Index group  N (%) | Prevalence of anaemia at baseline in index group, N (%) | Prevalence of anaemia at baseline in reference group, N (%) | Crude odds ratio (95% CI) | P value (chi squared test) |
|  | Leiden 85-plus Study | | 121/555 (21.8) | 58/121 (47.9) | 100/434 (23.0) | 3.08 (2.02 to 4.68) | <0.001 |
|  | LiLACS NZ (Māori) | | 51/207 (24.6) | 21/51 (41.2) | 24/156 (15.4) | 3.85 (1.90 to 7.81) | <0.001 |
|  | LilACS NZ (Non-Māori) | | 86/357 (24.1) | 37/86 (43.0) | 38/271 (14.0) | 4.63 (2.68 to 8.01) | <0.001 |
|  | Newcastle 85+ study | | 164/752 (21.8) | 80/164 (48.8) | 144/588 (24.5) | 2.94 (2.05 to 4.21) | <0.001 |
|  | TOOTH | | 16/345 (4.6) | 14/16 (87.5) | 153/329 (46.5) | 8.05 (1.80 to 36.0) | 0.001 |
| Abbreviations: OR, odds ratio; CI, confidence interval; eGFR, estimated glomerular filtration rate; CRP, C-reactive protein.  ^a^ Index group = iron deficiency, vitamin B12 deficiency, folate deficiency, low eGFR, high CRP; 1,2,3,4,5 combination of abnormal determinants; ≥2 combination of abnormal determinants.  ^b^ Population with anaemia within index group.  ^c^ Reference group = normal ferritin, normal vitamin B12, normal folate, normal eGFR, normal CRP; 0 combination of abnormal determinants; 0-1 combination of abnormal determinants. Population with anaemia within reference group.  ^d^ Crude (model 1). Results were presented as odds ratio with a 95% confidence interval.  ^e^ P value was derived from chi-square test except sum of combination of abnormal determinants.  ^f^ Iron deficiency was defined as ferritin <20 μg/L for men, <15 μg/L for women; vitamin B12 deficiency was <150 pmol/L; folate deficiency was serum folate level <7 nmol/L (Leiden 85-plus Study and TOOTH) or red blood cell folate <317 nmol/L (LiLACS NZ) and <340 nmol/L (Newcastle 85+ study); low eGFR was <45 mL/min/1.73 m^2^, eGFR was calculated using MDRD (Modification of Diet in Renal Disease) Study equation from the National Kidney Foundation; high CRP was >5 mg/L. Conversion factors: to convert serum vitamin B12 to picograms per milliliter, divide by 0.7378; to convert folate to nanograms per milliliter, divide by 2.265.  ^g^ All four studies included five determinants: iron, vitamin B12, folate deficiency, low eGFR, and high CRP.  ^h^ A population size of zero led to an inestimable odds ratio and p value. | | | | | | | |

| **Supplementary Table 4.** Cross-sectional Results: Single and a Combination of Determinants of Anaemia in Association with the Presence of Anaemia at Baseline in the Four Studies (Crude and Two Adjusted Models)^a^ | | | | | | | |
| --- | --- | --- | --- | --- | --- | --- | --- |
|  | |  | Leiden 85-plus Study (N=555) | LiLACS NZ  Māori (N=207) | LiLACS NZ  Non-Māori (N=357) | Newcastle 85+ study  (N=752) | TOOTH  (N=345) |
|  | |  | OR (95% CI) | OR (95% CI) | OR (95% CI) | OR (95% CI) | OR (95% CI) |
| Single Determinants^b^ |  | | | | | | |
| Iron Deficiency | | Model 1^c^ | 3.21 (1.69 to 6.11) | 0.89 (0.10 to 8.25) | 1.59 (0.59 to 4.32) | 3.66 (2.26 to 5.93) | 2.00 (1.11 to 3.60) |
|  | | Model 2^d^ | 2.95 (1.50 to 5.80) | 0.88 (0.09 to 8.99) | 1.59 (0.56 to 4.50) | 4.13 (2.52 to 6.78) | 2.15 (1.16 to 3.96) |
|  | | Model 3^e^ | 2.99 (1.51 to 5.89) | 0.87 (0.08 to 9.02) | 1.59 (0.56 to 4.52) | 4.19 (2.54 to 6.91) | 2.15 (1.16 to 3.97) |
| Vitamin B12 Deficiency | | Model 1 | 1.38 (0.84 to 2.25) | 0.75 (0.24 to 2.35) | 0.75 (0.34 to 1.63) | 0.84 (0.55 to 1.28) | 14.5 (1.87 to 112) |
|  | | Model 2 | 1.48 (0.89 to 2.47) | 0.89 (0.27 to 2.97) | 0.80 (0.35 to 1.79) | 0.88 (0.57 to 1.36) | 12.5 (1.59 to 98.3) |
|  | | Model 3 | 1.48 (0.88 to 2.46) | 0.90 (0.27 to 3.01) | 0.78 (0.35 to 1.77) | 0.91 (0.59 to 1.41) | 12.7 (1.61 to 99.5) |
| Folate Deficiency | | Model 1 | 2.76 (1.46 to 5.21) | 2.07 (1.03 to 4.17) | 1.43 (0.83 to 2.45) | 0.70 (0.28 to 1.77) | 0.51 (0.05 to 5.65) |
|  | | Model 2 | 2.06 (1.05 to 4.05) | 2.55 (1.20 to 5.42) | 1.26 (0.71 to 2.22) | 0.64 (0.24 to 1.69) | 0.19 (0.01 to 3.36) |
|  | | Model 3 | 2.05 (1.04 to 4.04) | 2.55 (1.20 to 5.41) | 1.28 (0.72 to 2.26) | 0.72 (0.27 to 1.89) | 0.17 (0.01 to 3.33) |
| Low eGFR^b^ | | Model 1 | 2.20 (1.43 to 3.40) | 5.14 (2.38 to 11.08) | 4.34 (2.30 to 8.19) | 2.97 (2.14 to 4.13) | 1.79 (0.72 to 4.45) |
|  | | Model 2 | 2.38 (1.51 to 3.76) | 5.73 (2.46 to 13.37) | 4.39 (2.23 to 8.66) | 3.16 (2.23 to 4.46) | 1.66 (0.65 to 4.22) |
|  | | Model 3 | 2.44 (1.54 to 3.88) | 6.96 (2.79 to 17.36) | 4.20 (2.12 to 8.32) | 3.06 (2.16 to 4.34) | 1.67 (0.65 to 4.25) |
| High CRP | | Model 1 | 2.19 (1.50 to 3.20) | 2.95 (1.45 to 5.97) | 2.68 (1.55 to 4.63) | 2.24 (1.60 to 3.12) | 2.15 (1.08 to 4.28) |
|  | | Model 2 | 1.97 (1.31 to 2.95) | 2.14 (0.99 to 4.61) | 2.64 (1.49 to 4.68) | 2.01 (1.43 to 2.83) | 1.94 (0.94 to 4.02) |
|  | | Model 3 | 2.02 (1.34 to 3.05) | 2.23 (1.02 to 4.90) | 2.72 (1.52 to 4.85) | 1.97 (1.40 to 2.78) | 1.95 (0.94 to 4.04) |
| Increase per Additional Abnormal Determinant^f^ | | Model 1 | 2.10 (1.67 to 2.63) | 2.21 (1.51 to 3.22) | 2.04 (1.49 to 2.80) | 2.10 (1.72 to 2.56) | 2.26 (1.52 to 3.35) |
|  |  | Model 2 | 2.04 (1.61 to 2.59) | 2.27 (1.50 to 3.41) | 1.94 (1.40 to 2.68) | 2.12 (1.73 to 2.59) | 2.16 (1.43 to 3.25) |
|  |  | Model 3 | 2.06 (1.62 to 2.63) | 2.36 (1.55 to 3.60) | 1.95 (1.41 to 2.70) | 2.11 (1.72 to 2.58) | 2.17 (1.44 to 3.27) |
| ≥2 Combination of Determinants^f^ | | Model 1 | 3.08 (2.02 to 4.68) | 3.85 (1.90 to 7.81) | 4.63 (2.68 to 8.01) | 2.94 (2.05 to 4.21) | 8.05 (1.80 to 36.0) |
|  | | Model 2 | 2.93 (1.87 to 4.58) | 4.16 (1.95 to 8.89) | 4.31 (2.43 to 7.63) | 3.12 (2.15 to 4.52) | 6.48 (1.42 to 29.4) |
|  | | Model 3 | 2.97 (1.89 to 4.65) | 4.60 (2.08 to 10.13) | 4.32 (2.43 to 7.69) | 3.12 (2.14 to 4.54) | 6.60 (1.45 to 30.1) |
| Abbreviations: OR, odds ratio; CI, confidence interval; eGFR, estimated glomerular filtration rate; CRP, C-reactive protein.  ^a^ LiLACS NZ contained two cohorts: Māori and non-Māori population; TOOTH: since not all determinants were collected at baseline, 3-year follow-up was defined as baseline, and 6-year follow-up as follow-up data.  ^b^ Iron deficiency was defined as ferritin <20 μg/L for men, <15 μg/L for women; vitamin B12 deficiency was <150 pmol/L; folate deficiency was serum folate level <7 nmol/L (Leiden 85-plus Study and TOOTH) or red blood cell folate<317 nmol/L (LiLACS NZ) and <340 nmol/L (Newcastle 85+ study); low eGFR was <45 mL/min/1.73 m^2^, eGFR was calculated using MDRD (Modification of Diet in Renal Disease) Study equation from the National Kidney Foundation; high CRP was >5 mg/L. Conversion factors: to convert serum vitamin B12 to picograms per milliliter, divide by 0.7378; to convert folate to nanograms per milliliter, divide by 2.265.  ^c^ Crude model. Results were presented as odds ratio with a 95% confidence interval.  ^d^ Adjusted for age (except Leiden 85-plus Study having all participants aged 85 years old), sex, institutionalisation (except TOOTH which served as an exclusion criteria) and smoking.  ^e^ Fully adjusted model: adjusted for age, sex, institutionalisation, smoking, and ≥2 multi-morbidity. Multi-morbidity was composed of stroke, coronary heart disease (CHD), cancer and diabetes. It was stratified into 0 to 1 or 2 and above as a binary variable. Leiden 85-plus Study: sex, institutionalisation, smoking and ≥2 multi-morbidity [stroke, coronary heart disease (CHD) excluding stroke, cancer, diabetes]; LiLACS NZ: age, sex, institutionalisation, smoking and ≥2 multi-morbidity [stroke (cerebrovascular accident (CVA), cardiovascular disease (CVD) excluding stroke, cancer, diabetes]; Newcastle 85+ study: age, sex, institutionalisation, smoking, ≥2 multi-morbidity (CVA, combined cardiac disease excluding CVA, cancer, diabetes); TOOTH: age, sex, smoking, ≥2 multi-morbidity (stroke , coronary heart disease (CHD), cancer, diabetes).  ^f^ All four studies included five determinants: iron, vitamin B12, folate deficiency, low eGFR, and high CRP. | | | | | | | |

| **Supplementary Table 5** The prevalence of 0, 1, 2, 3, 4 and 5 (out of 5) abnormal determinants for participants with and without anemia at baseline in four studies ^ab^ | | | | | | | | | | | | | | | |
| --- | --- | --- | --- | --- | --- | --- | --- | --- | --- | --- | --- | --- | --- | --- | --- |
|  | Leiden 85-plus Study  N=555 | |  | LiLACS NZ (Māori)  N=207 | |  | LilACS NZ (Non-Māori) N=356 | |  | Newcastle 85+ study  N=752 | |  | TOOTH  N=345 | |  |
|  | Anaemia at baseline^c^  n=158  N (%) | No anaemia at baseline^d^  n=397 | P value (Chi square test) ^e^ | Anaemia at baseline  n=45 | No anaemia at baseline  n=162 | P value (Chi square test) | Anaemia at baseline  n=75 | No anaemia at baseline  n=282 | P value (Chi square test) | Anaemia at baseline  n=224 | No anaemia at baseline  n=528 | P value (Chi square test) | Anaemia at baseline  n=167 | No anaemia at baseline  n=178 | P value (Chi square test) |
| **Sum of Abnormal Determinants** | | | | |  |  |  |  |  |  |  |  |  |  |  |
| 0 | 36 (22.8) | 190 (47.9) | <0.001 | 8 (17.8) | 63 (38.9) | <0.001 | 15 (20.0) | 106 (37.7) | <0.001 | 41 (18.3) | 221 (41.9) | <0.001 | 95 (56.9) | 134 (75.3) | <0.001 |
| 1 | 64 (40.5) | 144 (36.3) |  | 16 (35.6) | 69 (42.6) |  | 23 (30.7) | 126 (44.8) |  | 103 (46.0) | 223 (42.2) |  | 58 (34.7) | 42 (23.6) |  |
| 2 | 45 (28.5) | 58 (14.6) |  | 12 (26.7) | 24 (14.8) |  | 34 (45.3) | 42 (14.9) |  | 58 (25.9) | 74 (14.0) |  | 12 (7.2) | 2 (1.1) |  |
| 3 | 12 (7.6) | 4 (14.6) |  | 9 (20.0) | 6 (3.7) |  | 3 (4.0) | 6 (2.1) |  | 21 (9.4) | 9 (1.7) |  | 2 (1.2) | 0 |  |
| 4 | 1 (0.6) | 1 (0.3) |  | 0 | 0 |  | 0 | 0 |  | 1 (0.4) | 1 (0.2) |  | 0 | 0 |  |
| 5 | 0 | 0 |  | 0 | 0 |  | 0 | 1 (0.4) |  | 0 | 0 |  | 0 | 0 |  |
| **≥2 Combination of Determinants** | | | |  |  |  |  |  |  |  |  |  |  |  |  |
|  | 58 (36.7) | 63 (15.9) | <0.001 | 21 (46.7) | 30 (18.5) | <0.001 | 37 (49.3) | 49 (17.4) | <0.001 | 80 (35.7) | 84 (15.9) | <0.001 | 14 (8.4) | 2 (1.1) | 0.001 |
| ^a^ All variables were presented as number (percentage).  ^b^ All four studies included five determinants: iron, vitamin B12, folate deficiency, low eGFR, and high CRP.  ^c^ Within population with anaemia at baseline.  ^d^ Within population without anaemia at baseline.  ^e^ P value was derived from chi-square test. | | | | | | | | | | | | | | |  |

| **Supplementary Table 6.** Incidence of Anaemia from Age 85 Years Onwards, depending on the presence of single and a combination of determinants | | | | | | | | |
| --- | --- | --- | --- | --- | --- | --- | --- | --- |
|  | | | Index group  N (%)^a^ | | Incidence of anaemia in index group,  per 100 py (95%CI)^ab^ | Incidence of anemia in reference group, per 100 py (95%CI)^c^ | Crude HR  (95% CI)^d^ | P value (Cox regression)^e^ |
| **Iron deficiency^f^** | | |  | |  |  |  |  |
|  | Leiden 85-plus Study | | 17/360 (4.7) | | 11.8 (5.0 to 18.5) | 7.8 (2.3 to 13.3) | 1.45 (0.63 to 3.31) | 0.38 |
|  | Newcastle 85+ study | | 25/418 (6.0) | | 30.4 (19.6 to 41.2) | 10.8 (4.4 to 17.2) | 2.38 (1.34 to 4.26) | 0.003 |
|  | TOOTH | | 13/97 (13.4) | | 12.1 (5.3 to 18.9) | 13.9 (6.6 to 21.2) | 0.89 (0.31 ro 2.54) | 0.83 |
| **Vitamin B12 deficiency** | | |  | |  |  |  |  |
|  | Leiden 85-plus Study | | 53/360 (14.7) | | 8.8 (3.0 to 14.7) | 7.8 (2.3 to 13.3) | 1.12 (0.66 to 1.92) | 0.67 |
|  | Newcastle 85+ study | | 74/418 (17.7) | | 15.4 (7.7. to 23.1) | 10.9 (4.4 to 17.4) | 1.38 (0.88 to 2.16) | 0.16 |
|  | TOOTH | | 1/82 (1.2) | | 0 (-)^h^ | 13.9 (6.6 to 21.2) | 0.05 (-)^h^ | 0.70 |
| **Folate deficiency** | | |  | |  |  |  |  |
|  | Leiden 85-plus Study | | 19/360 (5.3) | | 25.6 (15.7 to 35.6) | 7.4 (2.1 to 12.7) | 3.01 (1.56 to 5.81) | 0.001 |
|  | Newcastle 85+ study | | 14/418 (3.3) | | 6.3 (1.4 to 11.3) | 11.9 (5.1 to 18.6) | 0.55 (0.14 to 2.23) | 0.40 |
|  | TOOTH | | 2/82 (2.4) | | 22.2 (13.0 to 31.5) | 13.5 (6.3 to 20.7) | 1.48 (0.20 to 10.90) | 0.70 |
| **Low eGFR** | | |  | |  |  |  |  |
|  | Leiden 85-plus study | | 54/361 (15.0) | | 8.2 (2.6 to 13.9) | 7.9 (2.4 to 13.4) | 1.02 (0.58 to 1.80) | 0.94 |
|  | Newcastle 85+ study | | 105/418 (25.1) | | 19.7 (11.0 to 28.4) | 9.4 (3.4 to 15.4) | 1.92 (1.30 to 2.83) | 0.001 |
|  | TOOTH | | 3/97 (3.1) | | 66.7 (50.7 to 82.7) | 12.7 (5.7 to 19.6) | 3.19 (0.98 to 10.36) | 0.05 |
| **High CRP** | | |  | |  |  |  |  |
|  | Leiden 85-plus study | | 97/361 (26.9) | | 11.3 (4.7 to 17.9) | 6.9 (1.8 to 12.1) | 1.58 (1.04 to 2.40) | 0.03 |
|  | Newcastle 85+ study | | 92/417 (22.1) | | 14.8 (7.3 to 22.4) | 10.9 (4.4 to 17.4) | 1.31 (0.86 to 2.00) | 0.21 |
|  | TOOTH | | 6/96 (6.3) | | 22.2 (13.0 to 31.5) | 13.3 (6.2 to 20.5) | 1.50 (0.46 to 3.92) | 0.50 |
| **Increase per Additional Abnormal Determinant^g^** | | | | | |  |  |  |
| Leiden 85-plus study | | 0 | | 180/361 (49.9) | 6.1 (1.3 to 10.9) | Reference | 1 |  |
|  | | 1 | | 128/361 (35.5) | 9.1 (3.2 to 15.0) | 6.1 (1.3 to 10.9) | 1.45 (0.93 to 2.26) | 0.10 |
|  | | 2 | | 48/361 (13.3) | 13.6 (6.4 to 20.9) |  | 2.09 (1.20 to 3.64) | 0.01 |
|  | | 3 | | 4/361 (1.1) | 14.3 (6.9 to 21.7) |  | 2.24 (0.54 to 9.27) | 0.27 |
|  | | 4 | | 1/361 (0.3) | 0 (-) |  | - | - |
|  | | 5 | | 0/361 (0) | 0 (-) |  | - | - |
| Newcastle 85+ study | | 0 | | 179/418 (42.8) | 7.0 (1.8 to 12.2) | Reference | 1 |  |
|  | | 1 | | 176/418 (42.1) | 13.6 (6.4 to 20.9) | 7.0 (1.8 to 12.2) | 1.84 (1.17 to 2.88) | 0.008 |
|  | | 2 | | 55/418 (13.2) | 22.1 (12.9 to 31.3) |  | 2.76 (1.60 to 4.75) | <0.001 |
|  | | 3 | | 8/418 (1.9) | 24.2 (14.6 to 33.9) |  | 3.07 (1.08 to 8.71) | 0.035 |
|  | | 4 | | 0/418 (0) | 0 (-) |  | - | - |
|  | | 5 | | 0/418 (0) | 0 (-) |  | - | - |
| TOOTH | | 0 | | 73/97 (75.3) | 12.5 (5.5 to 19.4) | Reference | 1 |  |
|  | | 1 | | 23/97 (23.7) | 16.2 (8.3 to 24.1) | 12.5 (5.5 to 19.4) | 1.24 (0.58 to 2.68) | 0.58 |
|  | | 2 | | 1/97 (1.0) | 66.7 (50.7 to 82.7) |  | 3.17 (0.43 to 23.5) | 0.26 |
|  | | 3 | | 0/97 (0) | 0 (-) |  | - | - |
|  | | 4 | | 0/97 (0) | 0 (-) |  | - | - |
|  | | 5 | | 0/97 (0) | 0 (-) |  | - | - |
|  | | |  | |  |  |  |  |
| **Combination of Determinants^g^** | | | | |  |  |  |  |
|  | Leiden 85-plus study | | 53/361 (14.7) | | 13.2 (6.1 to 20.4) | 7.2 (2.0 to 12.5) | 1.74 (1.07 to 2.85) | 0.03 |
|  | Newcastle 85+ study | | 63/418 (15.1) | | 22.4 (13.1 to 31.6) | 10.1 (3.9 to 16.3) | 1.99 (1.29 to 3.07) | 0.002 |
|  | TOOTH | | 1/97 (1.0) | | 66.7 (50.7 to 82.7) | 13.3 (6.2 to 20.5) | 3.00 (0.41 to 21.95) | 0.28 |
| Abbreviations: py, person-years; HR, hazard ratio; CI, confidence interval; eGFR, estimated glomerular filtration rate; CRP, C-reactive protein.  ^a^ Index group = iron, vitamin B12, folate deficiency, low eGFR, high CRP; 1,2,3,4,5 combination of abnormal determinants; ≥2 combination of abnormal determinants.  ^b^ Population who developed anaemia during follow-up within index group. Results were presented as per 100 person-years with 95% confidence interval.  ^c^ Reference group = normal ferritin, normal vitamin B12, normal folate, normal eGFR, normal CRP; 0 combination of abnormal determinants; 0-1 combination of abnormal determinants. Population who developed anaemia during follow-up within reference group.  ^d^ Crude (model 1) from cox regression. Results were presented as hazard ratio with a 95% confidence interval.  ^e^ P value was derived from cox regression.  ^f^ Iron deficiency was defined as ferritin <20 μg/L for men, <15 μg/L for women; vitamin B12 deficiency was <150 pmol/L; folate deficiency was serum folate level <7 nmol/L (Leiden 85-plus Study and TOOTH) and <340 nmol/L (Newcastle 85+ study); low eGFR was <45 mL/min/1.73 m2, eGFR was calculated using MDRD (Modification of Diet in Renal Disease) Study equation from the National Kidney Foundation; high CRP was >5 mg/L. Conversion factors: to convert serum vitamin B12 to picograms per milliliter, divide by 0.7378; to convert folate to nanograms per milliliter, divide by 2.265.  ^g^ LiLACS NZ did not have follow-up data for hemoglobin; TOOTH: since not all determinants were collected at baseline, 3-year follow-up was defined as baseline, and 6-year follow-up as follow-up data. All three studies included five determinants: iron, vitamin B12, folate deficiency, low eGFR, and high CRP.  ^h^ A population size of zero led to an inestimable hazard ratio and p value. | | | | | | | | |

| **Supplementary Table 7.** Prospective Results: Single and a Combination of Determinants at Baseline in Association with the Onset of Anaemia during Follow-up in Three Studies (Crude and Two Adjusted Models)^a^ | | | | |
| --- | --- | --- | --- | --- |
|  |  | Leiden 85-plus Study (N=361) | Newcastle 85+ study (N=418) | TOOTH  (N=97) |
|  | | HR (95%CI) | HR (95%CI) | HR (95%CI) |
| Single Determinants^b^ | |  |  |  |
| Iron Deficiency | Model 1^c^ | 1.45 (0.63 to 3.31) | 2.38 (1.34 to 4.26) | 0.89 (0.31 to 2.54) |
|  | Model 2^d^ | 1.47 (0.64 to 3.36) | 2.40 (1.34 to 4.30) | 0.88 (0.31 to 2.53) |
|  | Model 3^e^ | 1.48 (0.64 to 3.40) | 2.41 (1.35 to 4.32) | 0.88 (0.30 to 2.53) |
| Vitamin B12 Deficiency | Model 1 | 1.12 (0.66 to 1.92) | 1.38 (0.88 to 2.16) | 0.05 (-^g^) |
|  | Model 2 | 1.03 (0.59 to 1.79) | 1.33 (0.84 to 2.10) | -^g^ |
|  | Model 3 | 1.03 (0.59 to 1.79) | 1.35 (0.85 to 2.13) | - |
| Folate Deficiency | Model 1 | 3.01 (1.56 to 5.81) | 0.55 (0.14 to 2.23) | 1.48 (0.20 to 10.9) |
|  | Model 2 | 2.83 (1.45 to 5.51) | 0.54 (0.13 to 2.21) | 1.18 (0.11 to 12.6) |
|  | Model 3 | 2.84 (1.45 to 5.54) | 0.56 (0.14 to 2.30) | 1.20 (0.11 to 12.9) |
| Low eGFR | Model 1 | 1.02 (0.58 to 1.80) | 1.92 (1.30 to 2.83) | 3.19 (0.98 to 10.4) |
|  | Model 2 | 1.09 (0.61 to 1.94) | 2.00 (1.34 to 2.98) | 2.39 (0.68 to 8.40) |
|  | Model 3 | 1.09 (0.61 to 1.94) | 1.97 (1.32 to 2.94) | 2.41 (0.68 to 8.52) |
| High CRP | Model 1 | 1.58 (1.04 to 2.40) | 1.31 (0.86 to 2.00) | 1.50 (0.46 to 4.92) |
|  | Model 2 | 1.54 (1.00 to 2.39) | 1.28 (0.83 to 1.97) | 1.45 (0.42 to 4.95) |
|  | Model 3 | 1.56 (1.00 to 2.42) | 1.27 (0.83 to 1.96) | 1.45 (0.42 to 5.00) |
| Increase per Additional Abnormal Determinant^f^ | Model 1 | 1.34 (1.07 to 1.69) | 1.58 (1.26 to 1.97) | 1.38 (0.71 to 2.70) |
|  | Model 2 | 1.35 (1.06 to 1.73) | 1.57 (1.25 to 1.98) | 1.28 (0.65 to 2.53) |
|  | Model 3 | 1.35 (1.06 to 1.73) | 1.58 (1.25 to 1.98) | 1.28 (0.65 to 2.53) |
| ≥2 Combination of Determinants^f^ | Model 1 | 1.74 (1.07 to 2.85) | 1.99 (1.29 to 3.07) | 3.00 (0.41 to 22.0) |
|  | Model 2 | 1.86 (1.12 to 3.11) | 2.01 (1.30 to 3.12) | 1.97 (0.25 to 15.4) |
|  | Model 3 | 1.87 (1.12 to 3.12) | 2.02 (1.30 to 3.13) | 1.98 (0.25 to 15.5) |
| Abbreviations: HR, hazard ratio; CI, confidence interval; eGFR, estimated glomerular filtration rate; CRP, C-reactive protein.  ^a^ TOOTH: since not all determinants were collected at baseline, 3-year follow-up was defined as baseline, and 6-year follow-up as follow-up data.  ^b^ Iron deficiency was defined as ferritin <20 μg/L for men, <15 μg/L for women; vitamin B12 deficiency was <150 pmol/L; folate deficiency was serum folate level <7 nmol/L (Leiden 85-plus Study and TOOTH) or <340 nmol/L (Newcastle 85+ study); low eGFR was <45 mL/min/1.73 m^2^, eGFR was calculated using MDRD (Modification of Diet in Renal Disease) Study equation from the National Kidney Foundation. High CRP was >5 mg/L. Conversion factors: to convert serum vitamin B12 to picograms per milliliter, divide by 0.7378; to convert folate to nanograms per milliliter, divide by 2.265.  ^c^ Crude model. Results were presented as hazard ratio with a 95% confidence interval.  ^d^ Adjusted for age (except Leiden 85+ Study having all participants aged 85 years old), sex, institutionalisation (except TOOTH which served as an exclusion criteria) and smoking.  ^e^ Fully adjusted model: adjusted for age, sex, institutionalisation, smoking, and ≥2 multi-morbidity. Multi-morbidity was composed of stroke, coronary heart disease (CHD), cancer and diabetes. It was stratified into 0 to 1 or 2 and above as a binary variable. Leiden 85-plus Study: sex, institutionalisation, smoking and ≥2 multi-morbidity [stroke, coronary heart disease (CHD) excluding stroke, cancer, diabetes]; Newcastle 85+ study: age, sex, institutionalisation, smoking, ≥2 multi-morbidity (CVA, combined cardiac disease excluding CVA, cancer, diabetes); TOOTH: age, sex, smoking, ≥2 multi-morbidity (stroke , coronary heart disease (CHD), cancer, diabetes).  ^f^ All four studies included five determinants: iron, vitamin B12, folate deficiency, low eGFR, and high CRP.  ^g^ A population size of zero led to an inestimable hazard ratio. | | | | |

| **Supplementary Table 8** The prevalence of iron deficiency and the combination of abnormal determinants at baseline in the four studies using two different cut-offs for serum ferritin to define iron deficiency^ab^ | | | | | | | | | | |
| --- | --- | --- | --- | --- | --- | --- | --- | --- | --- | --- |
|  | **Serum Ferritin <15 μg/L (women), <20 μg/L (men)** | | | | | **Serum Ferritin <50 μg/L** | | | | |
|  | Leiden 85-plus (N=555) | LiLACS NZ  Māori  (N=207) | LiLACS NZ  Non-Māori (N=357) | Newcastle 85+  (N=752) | TOOTH  (N=345) | Leiden 85-plus (N=555) | LiLACS NZ  Māori  (N=207) | LiLACS NZ  Non-Māori (N=357) | Newcastle 85+  (N=752) | TOOTH  (N=345) |
| **Iron Deficiency** | |  |  |  |  |  |  |  |  |  |
|  | 41/554 (7.4) | 5/165 (3.0) | 20/298 (6.7) | 77/751 (10.3) | 56/344 (16.3) | 190/554 (34.3) | 30 /165 (18.2) | 67/298 (22.5) | 314 (41.8) | 162/344 (47.1) |
| **Sum of Combination of Abnormal Determinants**^c^ | | | |  |  |  |  |  |  |  |
| 0 | 226 (40.7) | 71 (34.3) | 121 (33.9) | 262 (34.8) | 229/345 (66.4) | 156 (28.1) | 61 (29.5) | 104 (29.1) | 161 (21.4) | 148 (42.9) |
| 1 | 208 (37.5) | 85 (41.1) | 150 (42.0) | 326 (43.4) | 100 (29.0) | 225 (40.5) | 84 (40.6) | 149 (41.7) | 324 (43.1) | 158 (45.8) |
| 2 | 103 (18.6) | 36 (17.4) | 76 (21.3) | 132 (17.6) | 14 (4.1) | 135 (24.3) | 43 (20.8) | 84 (23.5) | 209 (27.8) | 36 (10.4) |
| 3 | 16 (2.9) | 15 (7.2) | 9 (2.5) | 30 (4.0) | 2 (0.6) | 32 (5.8) | 19 (9.2) | 17 (4.8) | 49 (6.5) | 2 (0.6) |
| 4 | 2 (0.4) | 0 | 0 | 2 (0.3) | 0 | 7 (1.3) | 0 | 2 (0.6) | 9 (1.2) | 1 (0.3) |
| 5 | 0 | 0 | 1 (0.3) | 0 | 0 | 0 | 0 | 1 (0.3) | 0 | 0 |
| **Combination of Determinants (≥2)** | | |  |  |  |  |  |  |  |  |
|  | 121 (21.8) | 51 (24.6) | 86 (24.1) | 164 (21.8) | 16 (4.6) | 174 (31.4) | 62 (30.0) | 104 (29.1) | 267(35.5) | 39 (11.3) |
| ^a^ Variables were presented as number (percentage).  ^b^ LiLACS NZ contained two cohorts: Māori and non-Māori population.  ^c^ All four studies included five determinants: iron, vitamin B12, folate deficiency, low eGFR, and high CRP. Vitamin B12 deficiency was <150 pmol/L; folate deficiency was serum folate level <7 nmol/L (Leiden 85-plus Study and TOOTH) or red blood cell folate <317 nmol/L (LiLACS NZ), <340 nmol/L (Newcastle 85+ study); low eGFR was <45 mL/min/1.73 m2, eGFR was calculated using MDRD (Modification of Diet in Renal Disease) Study equation from the National Kidney Foundation. High CRP was >5 mg/L. | | | | | | | | | | |

| **Supplementary Table 9** Cross-sectional results: the association between iron deficiency, using two cut-offs for ferritin concentration, and the presence of anaemia at baseline in the four studies ^ab^ | | | | | | | | | | | |
| --- | --- | --- | --- | --- | --- | --- | --- | --- | --- | --- | --- |
|  |  | Serum Ferritin <15 μg/L (women), <20 μg/L (men) | | | | | Serum Ferritin <50 μg/L | | | | |
|  |  | Leiden 85-plus (N=555) | LiLACS NZ  Māori  (N=207) | LiLACS NZ  Non-Māori (N=357) | Newcastle 85+  (N=752) | TOOTH  (N=345) | Leiden 85-plus (N=555) | LiLACS NZ  Māori  (N=207) | LiLACS NZ  Non-Māori (N=357) | Newcastle 85+  (N=752) | TOOTH  (N=345) |
| **Iron Deficiency** | | |  |  |  |  |  |  |  |  |  |
|  | Model 1^c^ | 3.21 (1.69 to 6.11) | 0.89 (0.10 to 8.25) | 1.59 (0.59 to 4.32) | 3.66 (2.26 to 5.93) | 2.00 (1.11 to 3.60) | 1.70 (1.16 to 2.48) | 0.88 (0.33 to 2.34) | 0.93 (0.48 to 1.82) | 1.41 (1.03 to 1.94) | 1.38 (0.90 to 2.11) |
|  | Model 2^d^ | 2.95 (1.50 to 5.80) | 0.88 (0.09 to 8.99) | 1.59 (0.56 to 4.50) | 4.13 (2.52 to 6.78) | 2.15 (1.16 to 3.96) | 2.12 (1.40 to 3.19) | 1.12 (0.40 to 3.14) | 1.03 (0.52 to 2.07) | 1.69 (1.21 to 2.37) | 1.57 (1.01 to 2.46) |
|  | Model 3^e^ | 2.99 (1.51 to 5.89) | 0.87 (0.08 to 9.02) | 1.59 (0.56 to 4.52) | 4.19 (2.54 to 6.91) | 2.15 (1.16 to 3.97) | 2.12 (1.40 to 3.20) | 1.12 (0.40 to 3.14) | 1.03 (0.51 to 2.06) | 1.63 (1.16 to 2.30) | 1.57 (1.00 to 2.46) |
| **Increase per Additional Abnormal Determinant** | | | | |  |  |  |  |  |  |  |
|  | Model 1^c^ | 2.10 (1.67 to 2.63) | 2.21 (1.51 to 3.22) | 2.04 (1.49 to 2.80) | 2.10 (1.72 to 2.56) | 2.26 (1.52 to 3.35) | 1.95 (1.58 to 2.41) | 2.10 (1.45 to 3.04) | 1.76 (1.32 to 2.34) | 1.80 (1.50 to 2.16) | 1.71 (1.24 to 2.35) |
|  | Model 2 | 2.04 (1.61 to 2.59) | 2.27 (1.50 to 3.41) | 1.94 (1.40 to 2.68) | 2.12 (1.73 to 2.59) | 2.16 (1.43 to 3.25) | 2.03 (1.62 to 2.54) | 2.27 (1.51 to 3.42) | 1.74 (1.29 to 2.34) | 1.88 (1.56 to 2.27) | 1.71 (1.23 to 2.38) |
|  | Model 3 | 2.06 (1.62 to 2.63) | 2.36 (1.55 to 3.60) | 1.95 (1.41 to 2.70) | 2.11 (1.72 to 2.58) | 2.17 (1.44 to 3.27) | 2.05 (1.63 to 2.57) | 2.38 (1.57 to 3.63) | 1.75 (1.30 to 2.36) | 1.86 (1.53 to 2.25) | 1.72 (1.23 to 2.39) |
| **Combination of Determinants (≥2)** | | | |  |  |  |  |  |  |  |  |
|  | Model 1 | 3.08 (2.02 to 4.68) | 3.85 (1.90 to 7.81) | 4.63 (2.68 to 8.01) | 2.94 (2.05 to 4.21) | 8.05 (1.80 to 36.0) | 3.06 (2.08 to 4.50) | 3.30 (1.66 to 6.55) | 3.36 (1.98 to 5.71) | 2.10 (1.53 to 2.90) | 4.11 (1.89 to 8.95) |
|  | Model 2 | 2.93 (1.87 to 4.58) | 4.16 (1.95 to 8.89) | 4.31 (2.43 to 7.63) | 3.12 (2.15 to 4.52) | 6.48 (1.42 to 29.4) | 3.27 (2.16 to 4.95) | 3.94 (1.87 to 8.30) | 3.32 (1.01 to 5.79) | 2.20 (1.57 to 3.07) | 3.87 (1.75 to 8.58) |
|  | Model 3 | 2.97 (1.89 to 4.65) | 4.60 (2.08 to 10.13) | 4.32 (2.43 to 7.69) | 3.12 (2.14 to 4.54) | 6.60 (1.45 to 30.1) | 3.34 (2.20 to 5.06) | 4.55 (2.07 to 10.04) | 3.32 (1.90 to 5.80) | 2.16 (1.54 to 3.03) | 3.94 (1.77 to 8.75) |
| Results are presented as odds ratio (95% confidence interval)  ^a^ LiLACS NZ contained two cohorts: Māori and non-Māori population.  ^b^ All four studies included five determinants: iron, vitamin B12, folate deficiency, low eGFR, and high CRP vitamin B12 deficiency was <150 pmol/L; folate deficiency was serum folate level <7 nmol/L (Leiden 85-plus Study and TOOTH) or red blood cell folate<317 nmol/L (LiLACS NZ) and <340 nmol/L (Newcastle 85+ study); low eGFR was <45 mL/min/1.73 m^2^, eGFR was calculated using MDRD (Modification of Diet in Renal Disease) Study equation from the National Kidney Foundation; high CRP was >5 mg/L. Conversion factors: to convert serum vitamin B12 to picograms per milliliter, divide by 0.7378; to convert folate to nanograms per milliliter, divide by 2.265.  ^c^ Crude model. Results were presented as odds ratio with a 95% confidence interval.  ^d^ Adjusted for age (except Leiden 85-plus Study having all participants aged 85 years old), sex, institutionalisation (except TOOTH which served as an exclusion criteria) and smoking.  ^e^ Fully adjusted model: adjusted for age, sex, institutionalisation, smoking, and ≥2 multi-morbidity. Multi-morbidity was composed of stroke, coronary heart disease (CHD), cancer and diabetes. It was stratified into 0 to 1 or 2 and above as a binary variable. Leiden 85-plus Study: sex, institutionalisation, smoking and ≥2 multi-morbidity [stroke, coronary heart disease (CHD) excluding stroke, cancer, diabetes]; LiLACS NZ: age, sex, institutionalisation, smoking and ≥2 multi-morbidity [stroke (cerebrovascular accident (CVA), cardiovascular disease (CVD) excluding stroke, cancer, diabetes]; Newcastle 85+ study: age, sex, institutionalisation, smoking, ≥2 multi-morbidity (CVA, combined cardiac disease excluding CVA, cancer, diabetes); TOOTH: age, sex, smoking, ≥2 multi-morbidity (stroke , coronary heart disease (CHD), cancer, diabetes). | | | | | | | | | | | |

| **Supplementary Table 10** Meta-Analyses: Iron Deficiency (using two Cut-offs for ferritin concentration) ^a^, Combination of Determinants of Anaemia at Baseline in Association with Presence of Anaemia | | | | | | | | | |
| --- | --- | --- | --- | --- | --- | --- | --- | --- | --- |
|  |  | Serum Ferritin <15 μg/L (women), <20 μg/L (men) | | | | Serum Ferritin <50 μg/L | | | |
|  |  | N event/N total | N event/N total | OR | Weight | N event/N total | N event/N total | OR | Weight |
|  |  | (anaemia)^c^ | (no anaemia)^d^ | (95% CI) | % | (anaemia)^c^ | (no anaemia)^d^ | (95% CI) | % |
| **Iron Deficiency** | |  |  |  |  |  |  |  |  |
|  | Leiden 85-plus Study | 22/158 | 19/396 | 2.99 (1.51 to 5.89) | 23.4 | 68/158 | 122/396 | 2.12 (1.40 to 3.20) | 26.2 |
|  | LiLACS NZ (Māori) | 1/36 | 4/129 | 0.87 (0.08 to 9.02) | 2.7 | 6/36 | 24/129 | 1.12 (0.40 to 3.15) | 4.1 |
|  | LilACS NZ (Non-Māori) | 6/65 | 14/233 | 1.59 (0.56 to 4.52) | 11.9 | 14/65 | 53/233 | 1.03 (0.51 to 2.06) | 9.2 |
|  | Newcastle 85+ study | 44/224 | 33/527 | 4.19 (2.54 to 6.91) | 34.9 | 107/224 | 207/527 | 1.63 (1.16 to 2.30) | 38.2 |
|  | TOOTH | 35/166 | 21/178 | 2.15 (1.16 to 3.97) | 27.0 | 85/166 | 77/178 | 1.57 (1.01 to 2.46) | 22.3 |
| **Total (*I^2^*= 24%)** | |  |  | **2.76 (1.87 to 4.07)** | 100.0 | **(*I^2^*= 0%)** |  | **1.64 (1.32 to 2.02)** | 100.0 |
| **Increase per Additional Abnormal Determinant^b^** | | | |  |  |  |  |  |  |
|  | Leiden 85-plus Study | 36,64,45,12,1,0/158 | 190,144,58,4,1,0/397 | 2.06 (1.62 to 2.63) | 27.6 | 24,56,53,22,3,0/158 | 132,169,82,10,4,0/397 | 2.05 (1.63 to 2.57) | 26.7 |
|  | LiLACS NZ (Māori) | 8,16,12,9,0,0/45 | 63,69,24,6,0,0/162 | 2.36 (1.55 to 3.59) | 9.1 | 7,15,12,11,0,0/45 | 54,69,31,8,0,0/162 | 2.38 (1.56 to 3.63) | 7.8 |
|  | LilACS NZ (Non-Māori) | 15,23,34,3,0,0/75 | 106,127,42,6,1,0/282 | 1.95 (1.41 to 2.70) | 15.1 | 13,24,30,8,0,0/75 | 91,125,54,9,2,1/282 | 1.75 (1.30 to 2.36) | 15.5 |
|  | Newcastle 85+ study | 41,103,58,21,1,0/224 | 221,223,74,9,1,0/528 | 2.10 (1.72 to 2.58) | 38.6 | 19,98,75,27,5,0/224 | 142,226,134,22,4,0/528 | 1.86 (1.53 to 2.25) | 37.4 |
|  | TOOTH | 95,58,12,2,0,0/167 | 134,42,2,0,0,0/178 | 2.17 (1.44 to 3.27) | 9.6 | 63,74,27,2,1,0/167 | 85,84,9,0,0,0/178 | 1.72 (1.23 to 2.39) | 12.6 |
| **Total (*I^2^*= 0%)** | |  |  | **2.10 (1.85 to 2.38)** | 100.0 | **(*I^2^*= 0%)** |  | **1.91 (1.70 to 2.14)** | 100.0 |
| **≥2 Combination of Determinants^b^** | | |  |  |  |  |  |  |  |
|  | Leiden 85-plus Study | 58/158 | 63/397 | 2.97 (1.89 to 4.65) | 29.1 | 78/158 | 96/397 | 3.34 (2.20 to 5.07) | 27.1 |
|  | LiLACS NZ (Māori) | 9/45 | 3/162 | 4.60 (2.09 to 10.12) | 9.4 | 23/45 | 39/162 | 4.55 (2.07 to 10.03) | 10.2 |
|  | LilACS NZ (Non-Māori) | 10/75 | 8/282 | 4.32 (2.43 to 7.69) | 17.6 | 38/75 | 66/282 | 3.32 (1.90 to 5.80) | 17.9 |
|  | Newcastle 85+ study | 80/224 | 84/528 | 3.12 (2.14 to 4.54) | 41.4 | 107/224 | 160/528 | 2.16 (1.54 to 3.02) | 34.9 |
|  | TOOTH | 14/167 | 2/178 | 6.60 (1.45 to 30.03) | 2.6 | 30/167 | 9/178 | 3.94 (1.77 to 8.74) | 10.0 |
| **Total (*I^2^*= 0%)** | |  |  | **3.44 (2.70 to 4.38)** | 100.0 | **(*I^2^*= 26%)** |  | **3.00 (2.29 to 3.94)** | 100.0 |
| Abbreviations: OR, odds ratio; CI, confidence interval.  ^a^ Results of fully adjusted model (model 3): adjusted for age, sex, institutionalisation, smoking and ≥2 multi-morbidity.  ^b^ All four studies included five determinants: iron, vitamin B12, folate deficiency, low eGFR, and high CRP.  ^c^ Population with determinant within total anemic population.  ^d^ Population with determinant within total non-anemic population. | | | | | | | | | |

| **Supplementary Table 11** Prospective results: the association between iron deficiency, using two cut-offs for ferritin concentration, and the onset of anaemia at baseline in three studies | | | | | | | |
| --- | --- | --- | --- | --- | --- | --- | --- |
|  |  | Serum Ferritin <15 μg/L (women), <20 μg/L (men) | | | Serum Ferritin <50 μg/L | | |
|  |  | Leiden 85-plus Study (N=361) | Newcastle 85+ study (N=418) | TOOTH  (N=97) | Leiden 85-plus Study (N=361) | Newcastle 85+ study (N=418) | TOOTH  (N=97) |
|  |  | HR (95%CI) | HR (95%CI) | HR (95%CI) | HR (95%CI) | HR (95%CI) | HR (95%CI) |
| Iron Deficiency | |  |  |  |  |  |  |
|  | Model 1^b^ | 1.45 (0.63 to 3.31) | 2.38 (1.34 to 4.26) | 0.89 (0.31 to 2.54) | 1.11 (0.73 to 1.70) | 1.62 (1.11 to 2.36) | 0.85 (0.42 to 1.71) |
|  | Model 2^c^ | 1.47 (0.64 to 3.36) | 2.40 (1.34 to 4.30) | 0.88 (0.31 to 2.53) | 1.31 (0.85 to 2.01) | 1.82 (1.23 to 2.71) | 0.92 (0.44 to 1.91) |
|  | Model 3^d^ | 1.48 (0.64 to 3.40) | 2.41 (1.35 to 4.32) | 0.88 (0.30 to 2.53) | 1.31 (0.85 to 2.01) | 1.80 (1.21 to 2.67) | 0.92 (0.44 to 1.91) |
| Increase per Additional Abnormal Determinant^a^ | | | |  |  |  |  |
|  | Model 1^c^ | 1.34 (1.07 to 1.69) | 1.58 (1.26 to 1.97) | 1.38 (0.71 to 2.70) | 1.29 (1.04 to 1.59) | 1.52 (1.25 to 1.86) | 1.16 (0.65 to 2.08) |
|  | Model 2 | 1.35 (1.06 to 1.73) | 1.57 (1.25 to 1.98) | 1.28 (0.65 to 2.53) | 1.34 (1.08 to 1.67) | 1.56 (1.27 to 1.92) | 1.17 (0.66 to 2.10) |
|  | Model 3 | 1.35 (1.06 to 1.73) | 1.58 (1.25 to 1.98) | 1.28 (0.65 to 2.53) | 1.34 (1.08 to 1.67) | 1.55 (1.27 to 1.91) | 1.17 (0.66 to 2.10) |
| ≥2 Combination of Determinants | | |  |  |  |  |  |
|  | Model 1 | 1.74 (1.07 to 2.85) | 1.99 (1.29 to 3.07) | 3.00 (0.41 to 22.0) | 1.76 (1.11 to 2.71) | 1.83 (1.25 to 2.68) | 1.50 (0.36 to 6.27) |
|  | Model 2 | 1.86 (1.12 to 3.11) | 2.01 (1.30 to 3.12) | 1.97 (0.25 to 15.4) | 1.92 (1.23 to 2.99) | 1.90 (1.29 to 2.80) | 1.40 (0.33 to 5.89) |
|  | Model 3 | 1.87 (1.12 to 3.12) | 2.02 (1.30 to 3.13) | 1.98 (0.25 to 15.5) | 1.94 (1.25 to 3.01) | 1.89 (1.28 to 2.78) | 1.40 (0.33 to 5.94) |
| Abbreviations: HR, hazard ratio; CI, confidence interval.  ^a^ All three studies included five determinants: iron, vitamin B12, folate deficiency, low eGFR, and high CRP vitamin B12 deficiency was <150 pmol/L; folate deficiency was serum folate level <7 nmol/L (Leiden 85-plus Study and TOOTH) and <340 nmol/L (Newcastle 85+ study); low eGFR was <45 mL/min/1.73 m^2^, eGFR was calculated using MDRD (Modification of Diet in Renal Disease) Study equation from the National Kidney Foundation; high CRP was >5 mg/L.  ^b^ Crude model. Results were presented as hazard ratio with a 95% confidence interval.  ^c^ Adjusted for age (except Leiden 85-plus Study having all participants aged 85 years old), sex, institutionalisation (except TOOTH which served as an exclusion criteria) and smoking.  ^d^ Fully adjusted model: adjusted for age, sex, institutionalisation, smoking, and ≥2 multi-morbidity. Multi-morbidity was composed of stroke, coronary heart disease (CHD), cancer and diabetes. It was stratified into 0 to 1 or 2 and above as a binary variable. Leiden 85-plus Study: sex, institutionalisation, smoking and ≥2 multi-morbidity [stroke, coronary heart disease (CHD) excluding stroke, cancer, diabetes]; Newcastle 85+ study: age, sex, institutionalisation, smoking, ≥2 multi-morbidity (CVA, combined cardiac disease excluding CVA, cancer, diabetes); TOOTH: age, sex, smoking, ≥2 multi-morbidity (stroke, coronary heart disease (CHD), cancer, diabetes). | | | | | | | |

| **Supplementary Table 12**. Meta-Analyses: Iron Deficiency (using two Cut-offs for ferritin concentration) and Combination of Determinants of Anaemia in Association with Onset of Anaemia in Three Studies^abc^ | | | | | | | | | |
| --- | --- | --- | --- | --- | --- | --- | --- | --- | --- |
|  |  | Serum Ferritin <15 μg/L (women), <20 μg/L (men) | | | | Serum Ferritin <50 μg/L | | | |
|  |  | N event/N total | N event/N total | HR | Weight | N event/N total | N event/N total | HR | Weight |
|  |  | (anaemia)^d^ | (no anaemia)^e^ | (95% CI) | % | (anaemia)^d^ | (no anaemia)^e^ | (95% CI) | % |
| **Iron Deficiency** | |  |  |  |  |  |  |  |  |
|  | Leiden 85-plus Study | 6/98 | 11/262 | 1.48 (0.64 to 3.40) | 30.8 | 32/98 | 81/262 | 1.31 (0.85 to 2.02) | 39.1 |
|  | Newcastle 85+ study | 13/109 | 12/309 | 2.41 (1.35 to 4.32) | 47.6 | 57/109 | 114/309 | 1.79 (1.21 to 2.67) | 43.3 |
|  | TOOTH | 4/33 | 9/64 | 0.88 (0.30 to 2.53) | 21.6 | 13/33 | 29/64 | 0.92 (0.44 to 1.91) | 17.6 |
| **Total (*I^2^*= 32%)** | |  |  | **1.67 (0.96 to 2.90)** | 100.0 | **(*I^2^*= 29%)** |  | **1.41 (1.01** to **1.97)** | 100.0 |
| **Increase per Additional Abnormal Determinant^c^** | | |  |  |  |  |  |  |  |
|  | Leiden 85-plus Study | 41,37,18,2,0,0/98 | 139,91,30,2,1,0/263 | 1.35 (1.06 to 1.73) | 44.0 | 30,38,25,3,2,0/98 | 96,112,48,5,2,0/263 | 1.34 (1.08 to 1.67) | 43.9 |
|  | Newcastle 85+ study | 30,52,23,4,0,0/109 | 149,124,32,4,0,0/309 | 1.58 (1.25 to 1.98) | 50.3 | 14,49,35,9,2,0/109 | 97,134,67,10,1,0/309 | 1.55 (1.27 to 1.91) | 49.9 |
|  | TOOTH | 23,9,1,0,0,0/33 | 50,14,0,0,0,0/64 | 1.28 (0.65 to 2.53) | 5.7 | 15,16,2,0,0,0/33 | 32,30,2,0,0,0/64 | 1.17 (0.66 to 2.10) | 6.2 |
| **Total (*I^2^*= 0%)** | |  |  | **1.46 (1.24** to **1.71)** | 100.0 | **(*I^2^*= 0%)** |  | **1.43 (1.24** to **1.66)** | 100.0 |
| **≥2 Combination of Determinants^c^** | | |  |  |  |  |  |  |  |
|  | Leiden 85-plus Study | 20/98 | 33/263 | 1.87 (1.12 to 3.12) | 41.1 | 30/98 | 55/263 | 1.93 (1.24 to 3.00) | 41.7 |
|  | Newcastle 85+ study | 27/109 | 36/309 | 2.02 (1.30 to 3.13) | 56.3 | 46/109 | 78/309 | 1.89 (1.28 to 2.78) | 54.4 |
|  | TOOTH | 1/33 | 0/64 | 1.98 (0.25 to 15.53) | 2.6 | 2/33 | 2/64 | 1.40 (0.33 to 5.94) | 3.9 |
| **Total (*I^2^*= 0%)** | |  |  | **1.95 (1.40** to **2.71)** | 100.0 | **(*I^2^*= 0%)** |  | **1.88 (1.41** to **2.50)** | 100.0 |
| Abbreviations: HR, hazard ratio; CI, confidence interval.  ^a^ Iron deficiency was defined as ferritin <20 μg/L for men, <15 μg/L for women; vitamin B12 deficiency was <150 pmol/L; folate deficiency was serum folate level <7nmol/L (Leiden 85-plus Study and TOOTH) or <340 nmol/L (Newcastle 85+ Study); low eGFR was <45 mL/min/1.73 m^2^, eGFR was calculated using MDRD (Modification of Diet in Renal Disease) Study equation from the National Kidney Foundation; high CRP was >5 mg/L.  ^b^ Results of fully adjusted model (model 3): adjusted for age, sex, institutionalisation, smoking and ≥2 multi-morbidity. Multi-morbidity was composed of stroke, coronary heart disease (CHD), cancer and diabetes. It was stratified into 0 to 1 or 2 and above as a binary variable. Leiden 85-plus Study: sex, institutionalisation, smoking and ≥2 multi-morbidity [stroke, coronary heart disease (CHD) excluding stroke, cancer, diabetes]; Newcastle 85+ study: age, sex, institutionalisation, smoking, ≥2 multi-morbidity (CVA, combined cardiac disease excluding CVA, cancer, diabetes); TOOTH: age, sex, smoking, ≥2 multi-morbidity (stroke, coronary heart disease (CHD), cancer, diabetes).  ^c^ LiLACS NZ did not have follow-up data for hemoglobin; TOOTH: since not all determinants were collected at baseline, 3-year follow-up was defined as baseline, and 6-year follow-up as follow-up data. All three studies included five determinants: iron, vitamin B12, folate deficiency, low eGFR, and high CRP.  ^d^ Population with determinant within total anemic population during follow-up.  ^e^ Population with determinant within total non-anemic population during follow-up. | | | | | | | | | |
